# Chinese Patent Medicines in the Treatment of Coronavirus Disease 2019 (COVID-19) in China

**DOI:** 10.3389/fphar.2020.01066

**Published:** 2020-07-17

**Authors:** Wei Zhuang, Zheng Fan, Yanqi Chu, Haizheng Wang, Ying Yang, Li Wu, Nan Sun, Ge Sun, Yuqiao Shen, Xiaolan Lin, Guiming Guo, Shengyan Xi

**Affiliations:** ^1^ Department of Pharmacy, Xuanwu Hospital of Capital Medical University, Beijing, China; ^2^ Department of Clinical Pharmacy, Beijing Hospital of Traditional Chinese Medicine, Capital Medical University, Beijing, China; ^3^ Department of Pharmacy, Beijing Mentougou District Hospital of Traditional Chinese Medicine, Beijing, China; ^4^ Pharmacy Department, Beijing Mentougou Hospital, Beijing, China; ^5^ Pharmaceutical Department, Guangdong Hospital of Traditional Chinese Medicine, Guangzhou, China; ^6^ Department of Traditional Chinese Medicine, School of Medicine, Xiamen University, Xiamen, China

**Keywords:** COVID-19, Chinese Patent Medicines, pharmacological action, clinical application, Traditional Chinese Medicine

## Abstract

**Background:**

Coronavirus Disease 2019 (COVID-19) is an emerging and rapidly evolving disease, with no recommended effective anti-coronavirus drug treatment. Traditional Chinese Patent Medicines (CPMs) have, however, been widely used to treat COVID-19 in China, and a number of clinical practice results have shown them to have a significant role in its treatment. Consequently, numerous guidelines and expert consensus have recommended the use of CPMs to treat COVID-19.

**Aim of the Study:**

The objectives of this review are to provide up-to-date information on the pharmacology and clinical research on CPMs in the treatment of COVID-19, discuss the research findings, and to better guide clinical application and scientific research on CPMs in the treatment of COVID-19.

**Methods:**

The frequencies of CPM recommendations by guidelines and expert consensus for treatment of COVID-19 in China were ranked. This report identifies the top 10 CPMs, which include Huoxiang Zhengqi capsule (HXZQC), Lianhua Qingwen capsule (LHQWC), Jinhua Qinggan granule (JHQGG), Shufeng Jiedu capsule (SFJDC), Tanreqing injection (TRQI), Xiyanping injection (XYPI), Xuebijing injection (XBJI), Shenfu injection (SFI), Shengmai injection (SMI), and Angong Niuhuang pill (AGNHP). Relevant studies from 2000 to 2020 on these top 10 CPMs, covering usage, dosage, mechanism, curative effect, and precautions, were collected from pharmacopoeia, reports, and theses *via* library and digital databases (including PubMed, CNKI, Google Scholar, Web of Science, and Elsevier).

**Results:**

The properties of the top 10 CPMs included antiviral, antibacterial, anti-inflammatory, antipyretic and analgesic, anti-acute lung injury, anti-shock, immune regulation, and enhancement of pulmonary function. In addition, clinical research results and Chinese treatment data showed that the CPMs had good therapeutic efficacy in the treatment of COVID-19, and adverse reactions were minimal.

**Conclusions:**

Knowledge of the characteristics of the top 10 CPMs and precautions that should be taken may help clinicians to rationally improve therapeutic efficacy, and promote the role of Chinese Medicine in the control of the COVID-19 global epidemic.

## Introduction

COVID-19 is an emerging and rapidly evolving epidemic. The cumulative number of confirmed cases globally reached 1,040,772 on April 4, 2020, comprising 149,790 (14.39%) cured cases, and 55,698 (5.35%) deaths. The causative organism has been designated as the 2019 novel coronavirus (2019-nCoV). On January 30, 2020, the epidemic was declared a public health emergency of international concern by the World Health Organization (WHO) (Fisher and Heymann, 2020). On February 11, 2020, the WHO Director-General, Tedros Adhanom Ghebreyesus, announced that the disease caused by this new coronavirus was “COVID-19,” which is an acronym for “coronavirus disease 2019.” The virus seems to be highly contagious and had quickly spread to 119 countries and regions by March 12, 2020. The clinical spectrum of COVID-19 varies from asymptomatic or paucisymptomatic forms to clinical symptoms characterized by respiratory failure that necessitates mechanical ventilation and support in an intensive care unit (ICU), to multiorgan and systemic manifestations in terms of sepsis, septic shock, and multiple organ dysfunction syndromes (MODS) (Wu and McGoogan, 2020). Currently, there is no effective anti-coronaviral drug that is recommended for treatment of COVID-19, and no vaccine is available. There is no evidence supporting the efficacy of broad-spectrum antibiotics, gamma globulin, interferon, or corticosteroid therapy for COVID-19. Treatment is symptomatic, and oxygen therapy represents the major intervention for patients with severe infection. Mechanical ventilation may be necessary in cases of respiratory failure refractory to oxygen therapy (Huang et al., 2020; Zhang et al., 2020).

Traditional Chinese Medicine (TCM) has a long history and has played an important role in the prevention and treatment of serious epidemic diseases. During the development of the COVID-19 epidemic, more than 3,100 TCM medical staff were deployed to Hubei province, and TCM was included in the guidelines for the diagnosis and treatment of COVID-19. Currently, the total number of confirmed cases treated by TCM has reached 60,107 (Gao, 2020). The decoctions, CPMs, acupuncture, and other TCM treatments have been comprehensively used for treatment, mainly based on syndrome differentiation. Specific CPMs have been widely employed to treat COVID-19 with remarkable therapeutic effects (National Health Commission of the People’s Republic of China and National Administration of Traditional Chinese Medicine, 2020).

**Figure 1:**
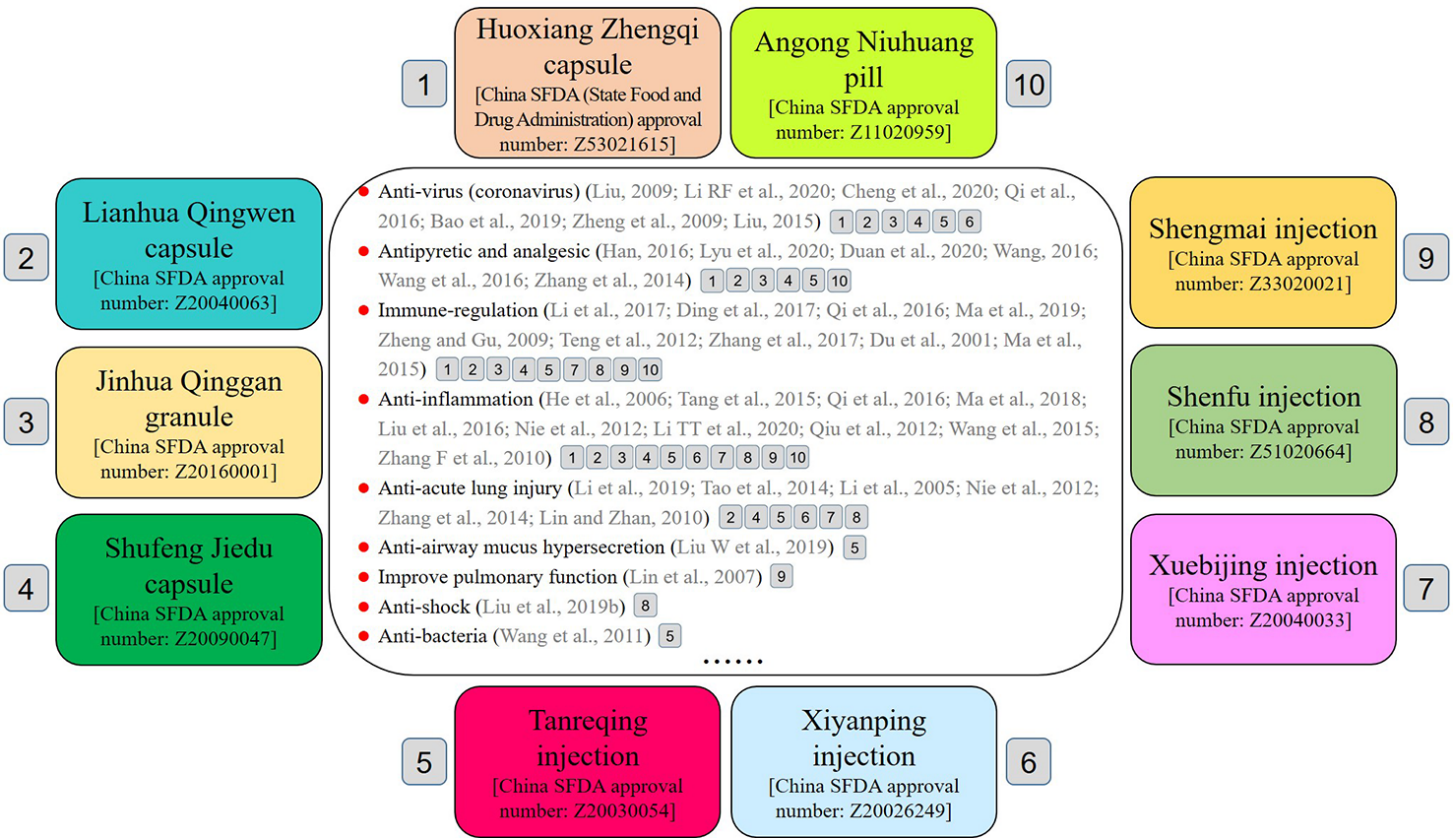
The top 10 CPMs for the treatment of COVID-19: 1) Huoxiang Zhengqi capsule (HXZQC), 2) Lianhua Qingwen capsule (LHQWC), 3) Jinhua Qinggan granule (JHQGG), 4) Shufeng Jiedu capsule (SFJDC), 5) Tanreqing injection (TRQI), 6) Xiyanping injection (XYPI), 7) Xuebijing injection (XBJI), 8) Shenfu injection (SFI), 9) Shengmai injection (SMI), and 10) Angong Niuhuang pill (AGNHP).

CPMs are approved by the National Drug Regulatory Authority of China and processed according to prescribed methods using Chinese herbal medicines as raw materials, guided by the theory of TCM. They are available in different dosage forms, such as pill, tablet, capsule, granule, or injection. The use of CPMs is guided by syndrome differentiation and overall analysis of signs and symptoms. Provinces of China have prepared therapeutic schedules for the treatment of COVID-19 based on actual conditions (see **Table 1**). Many guidelines and expert consensus in China have recommended using CPMs to treat COVID-19. In this article, we identify the top 10 recommended CPMs to treat COVID-19 (**Figure 1**). The list of CPMs includes Huoxiang Zhengqi capsule (HXZQC), Lianhua Qingwen capsule (LHQWC), Jinhua Qinggan granule (JHQGG), Shufeng Jiedu capsule (SFJDC), Tanreqing injection (TRQI), Xiyanping injection (XYPI), Xuebijing injection (XBJI), Shenfu injection (SFI), Shengmai injection (SMI), and Angong Niuhuang pill (AGNHP). Information on the drugs include the recommended guidelines, drug ingredients, indications, pharmacological research, clinical research, usage and dosage, adverse reactions, and precautions.

**Table 1:** Therapeutic regimens for COVID-19 in China.

Serial No.	Therapeutic regimen of COVID-19	Website	Date of issue
1	National Health Commission of the People’s Republic of China. Guideline on Diagnosis and Treatment of COVID-19 (Trial 7th edition)	http://www.nhc.gov.cn/yzygj/s7653p/202003/46c9294a7dfe4cef80dc7f5912eb1989.shtml	Mar. 03, 2020
2	Beijing Municipal COVID-19 TCM Preventive and Therapeutic Regime (Trial Version IV)	http://zyj.beijing.gov.cn/sy/tzgg/202003/t20200307_1682382.html	Mar. 06, 2020
3	Tianjin Municipal COVID-19 TCM Preventive and Therapeutic Regime (Trial Version III)	http://wsjk.tj.gov.cn/art/2020/2/21/art_70_71264.html	Feb. 20, 2020
4	Hebei Provincial COVID-19 TCM Therapeutic regime (Trial Version IV)	https://mp.weixin.qq.com/s/9QTqGDw6vkQyWX-VrjulqQ	Feb. 13, 2020
5	Gansu Provincial TCM Preventive and Therapeutic Regime of Novel Coronavirus Infected Pneumonia (Trial Version II)	http://wsjk.gansu.gov.cn/file.jsp?contentId=83488	Feb. 01, 2020
6	Guangdong Provincial COVID-19 TCM Therapeutic Regime (Trial Version II)	http://szyyj.gd.gov.cn/zwgk/gsgg/content/post_2902010.html	Feb. 18, 2020
7	Shaanxi Provincial TCM Therapeutic Regime of Novel Coronavirus Infected Pneumonia (Trial Version II)	http://sxwjw.shaanxi.gov.cn/art/2020/2/2/art_10_67602.html	Feb. 02, 2020
8	Hunan Provincial TCM Diagnosis And Treatment Scheme of Novel Coronavirus Infected Pneumonia (Trial Version III)	http://tcm.hunan.gov.cn/tcm/xxgk/tzgg/202002/t20200203_11168981.html	Feb. 03, 2020
9	Jilin Provincial TCM Therapeutic Regime of Novel Coronavirus Infected Pneumonia (Trial Version I)	http://jltcm.jl.gov.cn/tzgg/xgdt/202001/t20200126_6654768.html	Jan. 26, 2020
10	Technical Guidelines of Sichuan Province on TCM Prevention and Control of COVID-19	http://wsjkw.sc.gov.cn/scwsjkw/zcwj11/2020/2/6/ac6fea21a3ad490aa0a73c9d70004ad6.shtml	Feb. 05, 2020
11	Shanghai Municipal COVID-19 TCM Diagnosis And Treatment Scheme (Trial Version II)	http://wsjkw.sh.gov.cn/zyygz2/20200224/a1f1aab9745e4490867cb4aaf40eaad0.html	Feb. 24, 2020
12	Jiangxi Provincial COVID-19 TCM Preventive and Therapeutic Regime (Trial Version III)	http://www.jxhfpc.gov.cn/doc/2020/02/21/140518.shtml	Feb. 21,2020
13	COVID-19 TCM Therapeutic Regime of Guangxi Zhuang Autonomous Region (Trial Version III)	http://wsjkw.gxzf.gov.cn/zwgk/zfxxgkml/wsjszh/zyzy/2020/0224/1752.html	Feb. 24, 2020
14	TCM Preventive and Therapeutic Regime for Novel Coronavirus Infected Pneumonia of the Xinjiang Uygur Autonomous Region	http://www.xjhfpc.gov.cn/info/2074/17765.htm	Jan. 30, 2020
15	Hainan Provincial COVID-19 TCM Preventive and Therapeutic Regime (Trial Version III)	http://wst.hainan.gov.cn/swjw/xxgk/0200/0202/202003/t20200305_2756534.html	Feb. 14, 2020
16	Heilongjiang Provincial COVID-19 TCM Preventive and Therapeutic Regime (Version III)	http://www.hljdaily.com.cn/article/90/154485.html	Feb. 26, 2020
17	Guizhou Provincial COVID-19 TCM Preventive and Therapeutic Reference Regime (Version II)	http://atcm.guizhou.gov.cn/xwzx/zyyw/202002/t20200219_50116162.html	Feb. 19, 2020
18	Shanxi Provincial of TCM Preventive and Therapeutic Regime of Novel Coronavirus Infected Pneumonia (For Trial Implementation)	http://www.sx.chinanews.com/news/2020/0201/162758.html	Feb. 01, 2020
19	Jiangsu Provincial COVID-19 TCM Diagnosis and Intervention Regime (Trial Version III)	http://www.jstcm.com/article_info.asp?id=10042	Feb. 18, 2020
20	COVID-19 TCM Diagnosis and Treatment Scheme of the Nei Monggol Autonomous Region (Trial Version II)	http://wjw.nmg.gov.cn/doc/2020/02/18/292482.shtml	Feb. 14,2020
21	Liaoning Provincial TCM Diagnosis and Treatment Scheme of Novel Coronavirus Infected Pneumonia (Trial Version II)	http://wsjk.ln.gov.cn/wst_zdzt/xxgzbd/tzgg/202002/t20200203_3733244.html	Feb. 03, 2020
22	Anhui Provincial COVID-19 TCM Therapist Consensus	http://wjw.ah.gov.cn/ahtcm/NewsDetail.aspx?id=987	Feb. 18, 2020
23	Shandong Provincial TCM Diagnosis and Treatment Scheme of Novel Coronavirus Infected Pneumonia	http://wsjkw.shandong.gov.cn/ztzl/rdzt/qlzhfkgz/fkdt/202002/t20200201_2513391.html	Jan. 31, 2020
24	TCM Preventive and Therapeutic Regime for Novel Coronavirus Infected Pneumonia of the Ningxia Hui Autonomous Region (For Trial Implementation)	http://wsjkw.nx.gov.cn/info/1040/13360.htm	Jan. 28, 2020
25	TCM Diagnosis and Treatment Scheme and Preventive Scheme for Novel Coronavirus Infected Pneumonia of Tongji Hospital, Tongji Medical College, Huazhong University of Science & Technology	https://www.tjh.com.cn/html/2020/0208/28991.shtml	Feb. 08,2020

## Relevant Information on the Clinical Application of HXZQC

### Recommended Therapeutic Regimens

HXZQC has been recommended in 20 therapeutic regimens for COVID-19 in China (see detailed information in **Tables 1** and **2**).

**Table 2:** List of recommended CPMs in therapeutic regimens for COVID-19.

Drug name	Therapeutic regimens of COVID-19	Number of “therapeutic regimens”
AGNHP	2-13, 15‑19, 21‑23, 25	21
HXZQC	1, 4-8, 10‑16, 18, 19, 21‑25	20
XBJI	1-4, 6, 7, 10‑13, 15, 16, 17‑25	20
LHQWC	1, 2, 4-7, 11‑16, 18, 19, 21‑25	19
SFI	1-4, 6, 10‑13, 15, 16, 18‑25	19
SMI	1, 3, 6, 7, 10‑13, 15, 16, 18‑25	18
SFJDC	1, 4‑7, 11‑13, 16, 18, 19, 21‑24	15
XYPI	1, 3, 6, 7, 10‑13, 15, 20‑25	15
JHQGG	1, 2, 4‑7, 11, 12, 18, 21, 22, 24, 25	13
TRQI	1-3, 6, 7, 11‑13, 16, 19, 21, 22	12

### Ingredients of HXZQC

*Pogostemon cablin* (Blanco) Benth. (Guanghuoxiang), *Atractylodes macrocephala* Koidz. (Baizhu), *Magnolia officinalis* Rehder & E.H.Wilson (Houpo), *Pinellia ternata* (Thunb.) Makino (Banxia), *Perilla frutescens* (L.) Britton (Zisu), *Angelica dahurica* (Hoffm.) Benth. & Hook.f. ex Franch. & Sav. (Baizhi), *Citrus × aurantium* L. (Chenpi), *Poria cocos* (Schw.) Wolf (Fuling), *Platycodon grandiflorus* (Jacq.) A.DC. (Jiegeng), *Glycyrrhiza uralensis* Fisch. ex DC. (Gancao), *Ziziphus jujuba* Mill. (Dazao), *Areca catechu* L. (Binglang), and *Zingiber officinale* Roscoe (Shengjiang). Basic information on HXZQC is provided in the **Supplementary Table**.

### Indications for the Treatment of COVID-19 With HXZQC

HXZQC is used for cold outside and inside damp indications during the clinical observation period of COVID-19 and early stage of the disease (mild case). The indicative symptoms include weakness, headache and dizziness, abdominal fullness and distention, vomiting, and diarrhea.

### Progress of Pharmacological Research on HXZQC

Modern pharmacological studies have found that HXZQC has antiviral, anti-inflammatory, and immune regulatory activities, improves gastrointestinal discomfort and other properties (see **Table 3**).

**Table 3:** Pharmacological functions and clinical research on top 10 CPMs for the treatment of COVID-19.

Drug name	Pharmacological action	Mechanism	Clinical application	Therapeutic efficacy
HXZQC	Regulate the immunity and improve the gastroenteric function	Inhibits LPS and epithelial barrier disorder, stipulate expression of proinflammatory cytokine of macrophage (Wang et al., 2012). Improve thymus coefficient, spleen coefficient, immunoglobulin G (IgG) level of the mice (Li et al., 2017). Regulates effect of the balance of CD4^+^ and CD8^+^ T lymphocytes, and reduction of IL-2, IL-10, IL-1β and TNF-α level (He et al., 2006; Zong et al., 2015). Improves serum NO level of rats, reduces concentration of 5-HT, and downregulates the level of plasma motilin and colonic somatostatin (Huang et al., 2016).	Children with rotavirus enteritis	Shortens anti-diarrheal time and total course of time in treating children with rotavirus enteritis (Ma and Wang, 2012).
	Anti-virus	Inhibits Avian Influenza Virus H5N1 regulating the gastrointestinal tract (Liu, 2009).	Influenza	Extends relief time of fever symptom, relieves time of muscle aches and relieves time of fatigue (Han, 2016).
	Inhibition effect of vibrio parahaemolyticus, Candida albicans, staphylococcus aureus and diplococcus pneumonia (Zhang, 2013)	–	Cold	Relieves fever, nasal congestion, running nose, spontaneous sweating, headache, cough and spitting, fatigue and weakness, body ache and other cold symptoms (Wu, 2010; Zhao et al., 2017).
			Acute gastro-enteritis	Improves total response rate and clinical symptoms.
LHQWC	Anti-virus	Inhibits proliferation of influenza virus and regulates immune response to viral infection (Ding et al., 2017; Yao et al., 2020). Inhibits SARS-CoV-2 replication, affects virus morphology and exerts anti-inflammatory activity *in vitro* (Li R. F. et al., 2020).	COVID-19	Improves the fever, weakness, cough, short breath, chest distress, anorexia and other clinical symptoms of COVID-19, reduces the ratio of common to severe (Cheng et al., 2020; Hu et al., 2020; Lyu et al., 2020). Lianhua Qingwen granule combined with Abidole can effectively relieve clinical symptoms of mild COVID-19 patients, regulate expression of related inflammatory factors, improve the curative effect and reduce the rate of severe illness (Liu et al., 2020; Yu P. et al., 2020).
	Anti-acute lung injury	Inhibits expression and secretion level of MCP-1, reduces infiltration of mononuclear macrophages (Li et al., 2019). Reduces LDH and MDA level, increases content of GSH-Px, and relieves the exudation of inflammatory cells in the alveolar cavity (Ping et al., 2016). Downregulates expression of IKK/IκB/NF-κB signaling pathway (Cui et al., 2016). Reduces protein expression and mRNA expression of inflammatory cytokines in lung tissues through reducing content of inflammatory cytokines in mice blood (Tang et al., 2015).	H1N1	Improves cough, sore throat, body ache and other symptoms of the patients infected with H1N1 virus, and reduces the duration of fever (Duan et al., 2011; Zhao P. et al., 2014).
			Influenza	LHQWC has better total response rate, symptom improvement rate and body temperature recovery rate than the control group (Wang et al., 2019).
			URI	Improves the patients’ nasal congestion, fever, headache, sore throat, weakness, aches in the limbs, intolerance of cold and other clinical symptoms (Li, 2019).
			Chronic obstructive pulmonary	Improves the condition of the patients with AECOPD, and reduces release of inflammatory mediators (Dong et al., 2014).
JHQGG	Anti-virus	Decreases average level of CRP and IFN-γ in serum of the influenza patients, and decreases inflammatory response (Qi et al., 2016).	COVID-19	Alleviates symptoms of fever, cough, fatigue and sputum (Duan et al., 2020).
			H1N1	Shortens antipyretic time (Wang C. et al., 2011).
			Influenza	Reduces the serum levels of cytokines and improve their immune function (Li et al., 2013; Qi et al., 2016).
SFJDC	Anti-virus	Improves mice pneumonia symptoms caused by influenza virus, reduce lung index of the mice infected with H1N1, and significantly reduces mortality rate of the infected animals (Liu et al., 2010; Lv et al., 2013; Qiu et al., 2014; Bao et al., 2019).	Acute URI + fever	Improves respiratory symptoms (Wang and Qiu, 2018)
	Anti-inflammation	Reduces the WBC count, and reduces the serum transcription factor NF-κB, chemokine MCP-1, inflammatory mediator BK and COX-2 level (Ma et al., 2018).	CAP	Shortens recover time of multiple symptoms and signs such as fever, reduces levels of PCT, CRP and WBC and other indicators (Wang, 2016).
	Immune-regulation	Reduces levels of B lymphocytes, CD8+ proportion, IL-1α, IL-1β, IL-2, IgM, IgG, etc., reduces quality of thymus, spleen and lung of pneumonia mice, and increase CD4+/CD8+ and NK cell proportion (Ma et al., 2019).	AECOPD	Reduces levels of IL-8, TNF-α, CRP and PCT (Yu H. X. et al., 2020) and increases arterial blood gas PaO_2_ (Wang, 2015).
	Anti-acute lung injury	Inhibition of the MAPK/NF-κB signaling pathway, and down-regulation of NF-κB mRNA expression (Tao et al., 2014). The action might be closely related with ERK signaling pathway (Li et al., 2017).	Bacterial acute bronchitis and Pneumonia	Shortens recovery time of body temperature, duration of cough and the course of treatment, and increases oxygen index (Wang et al., 2014).
TRQI	Anti-virus	Inhibition of intracellular proliferation and enhancement of body immunity of mice infected with influenza virus (Zheng and Gu, 2009). Destroys MRSA biofilm, and induces its death, and when in combined use with vancomycin or linezolid below the MIC concentration (Yang et al., 2018).	Viral pneumonia	A systematic review: TRQI had advantages in response rate of treatment, average length of stay (Pan, 2016).
	Anti-bacteria	Destroys the bacterial biofilm (Wang Y. et al., 2011).	Acute bronchitis	Improves response rate, reduce fever, cough, crackles and X-ray shadow absorption (Wang et al., 2016)
	Anti-inflammation	Inhibits release of inflammatory factors such as TNF-α, IL-6 and NO, and inhibits airway inflammation caused by LPS through MAPK/NF-B pathway (Liu et al., 2016).	Acute attack of chronic bronchitis	Improves clinical symptoms (Gao et al., 2019).
	Anti-airway mucus hypersecretion	Regulates the IL-17 signaling pathway and its downstream protein MUC5AC (Liu W. et al., 2019).	CAP	Improves clinical effect and the symptom of cough with expectoration, shortens the duration of fever and promotes shadow absorption on chest radiography and the hemogram recovery (Jiang et al., 2009).
	Anti-acute lung injury	Improves blood flow status of capillaries in the alveolar walls while repressing LPS-induced inflammatory cascade (Li et al., 2005).	Tuberculosis accompanied by infection	A systematic review: TRQI might have the same overall effect with some antibacterial drugs in treating patients with tuberculosis accompanied by lung infection (Lian et al., 2018).
			AECOPD	Reduction of the patients’ serum IL-8 and NE level, and improvement of airway inflammation reaction and mucus hypersecretion (Li et al., 2010). A systematic review: improves clinical effect and lung function of AECOPD patients, reduces pCO_2_, and shortens the length of stay (Zhong et al., 2010).
XYPI	Anti-virus	Inhibits proliferation of human rhinovirus in mice (Liu, 2015).	Viral pneumonia	Increases the cure rate, and improve the symptoms and signs (Li, 2015; Qi et al., 2018)
	Anti-acute lung injury	Inhibits release of proinflammatory cytokines such as IL-10, IL4, etc., and could promote the proinflammatory cytokines/anti-inflammatory cytokines to tend to be balanced, and inhibit excess anti-inflammatory responses during the course of acute lung injury (Nie et al., 2012).	Severe pneumonia	Shortens the course of disease, improves the treatment efficiency, reduces incidence rate of antibiotic resistance, reduces occurrence of double infection, further improves the prognosis and reduces the mortality rate (Zhang and Wang, 2015). Reduces leukocytes, improve oxygen index, lower CPIS score, promotes absorption of pulmonary inflammation, shortens the duration of mechanical ventilation and length of stay in ICU, and improves clinical effect (Yang et al., 2014).
	Inhibition effect of staphylococcus aureus and pneumonia streptococcus (Yu et al., 2009)	–	Upper respiratory infection	Improves symptoms of the patients (Liu and Li, 2015).
XBJI	Anti-inflammation	Downregulates expression of inflammatory cytokines stimulated by Pam3CSK4 and activating MAPK, PI3K/Akt and other pathways (Li T. T. et al., 2020). Reduces TNF-α, IL-6 and IL-10 level of mice with sepsis, prevents the neutrophils from infiltrating the lung and kidney, inhibit Th1/Th2, Th17 and Tregs balance (Zhang et al., 2006; Chen et al., 2018).	COVID-19	Improves the inflammatory markers and prognosis of severe COVID-19 patients (Wen et al., 2020).
			SIRS	Expression of CD4^+^, CD4^+^/CD8^+^, CD14^+^/HLA-DR increased, and improves systematic status of the SIRS patients (Zhao W. et al., 2014).
			Severe pneumonia	Reduces the level of inflammatory factors, improves the total treatment efficiency (Qi et al., 2011), reduces infectious indicators and the average length of stays (Zhu et al., 2014).
	Anti-acute lung injury	Reduces TNF-α level, alleviates the degree of pulmonary tissue edema and inflammatory cell infiltration (Zhang et al., 2014).	SCAP	Improves primary endpoint-pneumonia severity index, reduces mortality rate in 28 days, and shortens the duration of mechanical ventilation (Song et al., 2019).
	Immune-regulation	Blocks p-38 MAPK and NF-κB 65 pathways (Liu et al., 2014); reduces IL-1, IL-6 and TNF-α level, improves CD4^+^/CD8^+^ T lymphocyte ratio and NK cell relative activity (Teng et al., 2012).	AECOPD	Lowers the inflammatory indicators, improve cough, expectoration, short breath and other clinical symptoms, and shortens their length of stay (Chen et al., 2011; Zhu et al., 2019).
	Anti-oxidation	Improves activity of SOD, reduce ROS level (Jin et al., 2018). Downregulates MDA (Luo and Zhou, 2017).	Sepsis	Reduces mortality rate of sepsis patients in 28 days, the APACHE-II and body temperature (Li et al., 2018).
SFI	Anti-acute lung injury	Increases the wet/dry weight ratio of lung tissues, neutrophil ratio in BALF, protein content, lung tissue MDA and serum NO (Lin and Zhan, 2010). Reduces activation of NF-κB of lung tissue (Ai et al., 2006). Reduces expression level of p65, P50 mRNA and protein in lung tissues and TNF-α level in serum (Liu et al., 2019a).	Sepsis	Lowers IL-6 level, regulate balance between pro-inflammatory factors and anti-inflammatory factors (Qiu et al., 2012). Increases CD4^+^ and CD8^+^T cell counts in peripheral blood and upregulated HLA-DR expression in monocytes (Zhang et al., 2017).
	Anti-shock	Increases content of ATP and taurine, and reduces content of AMP in the heart (Liu et al., 2019b).	Severe pneumonia of elderly	Decreases level of TNF-α, IL-6 and IL-8 (Lv et al., 2017).
			Acute lung injury	Improves respiratory rate, oxygen index, and lowers the ICAM-1, ET-1 and NO level (Ma et al., 2019).
			Respiratory failure	Improves serum prealbumin, oxygen index, shortens the duration of mechanical ventilation (Li, 2013)
SMI	Improve pulmonary function	Raises NO level, dropping oxygen free radical levels and decreases lipid peroxidation (Lin et al., 2007). Lowers expression of NF-κB and activity of iNOS in lung tissues (Liu et al., 2009).	COPD	Improves pulmonary function index, blood gas index, IgG index and disappearance time of lung rale (Huang et al., 2019).
	Anti-inflammation	Inhibits expression of ICAM-1 and VCAM-1 (Liu et al., 2015). Inhibits generation of inflammatory cytokines of ischemia-reperfusion rats, lowers expression level of TNF-α, IL-6, IL-8, etc. (Wang et al., 2015)		
	Immune-regulation	Inhibits monocyte MCP-1 (Liu et al., 2015). Increases the content of serum immunoglobulin IgG and the number of T cells, enhances the phagocytic function of macrophages (Du et al., 2001).	Prevent inflammatory response	Improves microcirculation, protect the organ functions, and prevents further occurrence and development of systemic inflammatory response syndrome (Guo et al., 2004).
	Improve pulmonary function	Raises NO level, dropping oxygen free radical levels and decreases lipid peroxidation (Lin et al., 2007). Lowers expression of NF-κB and activity of iNOS in lung tissues (Liu et al., 2009).		
AGNHP	Anti-inflammation	Inhibits release of superoxide radical; reverses changes in cortical monoamine neurotransmitters (Zhang F. et al., 2010; Zhu and Sun, 2014). Lowers serum LPS level and lung myeloperoxidase (MPO) content (Zhang et al., 2009). Lowers total LDH activity, and changes percentage of isomerase (Tang et al., 2005).	Hyperpyrexia, coma caused by severe infectious diseases	Promotes consciousness, improves the neurologic function (Feng and Yang, 2015), shortens average defervescence time (Long and Wu, 2014) and moderates effect on Th1/Th2 (Ma and Zhou, 2015).
			Viral encephalitis	Reduces body temperature, avoids convulsion, promotes consciousness, and alleviates cerebral edema and brain cell damage (Zhang and Dong, 2014).
	Neuroprotective effect	Regulates Th17/Treg balance, inhibits chronic inflammation, reduces plaque collagen fibers, and reduces inflammatory cells infiltration (Fan et al., 2020)	Pneumonia	Reduces PCT and improves immune function (Zhuo and Wen, 2017).
	Antipyretic and analgesic	–	ACI intracerebral hemorrhage	Neuroprotective effect (Han et al., 2019).

Research by Zhonghua Liu et al. (Liu, 2009) revealed that HXZQC inhibited the lung index of mice infected with avian influenza virus (AIV) H5N1, reduced the development of lung disease, and enhanced the antiviral capacity of mice infected with AIV. The death rate of the infected mice was reduced through regulation of the gastrointestinal tract and strengthening of the stomach Qi. Hongkun Zhang (Zhang, 2013) found that HXZQC inhibited growth of *Vibrio parahaemolyticus*, *Candida albicans*, *Staphylococcus aureus*, and *Streptococcus pneumoniae*. Research by Wang et al. (2012) found that HXZQC inhibited lipopolysaccharide (LPS)-stimulated expression of proinflammatory cytokines by macrophages and inhibited epithelial barrier disorder induced by interferon-γ (IFN-γ), regulating immunity and improving gastroenteric function. Research by Chunyuan Li et al. (Li et al., 2017) showed that HXZQC significantly improved the thymus coefficient, spleen coefficient and immunoglobulin G (IgG) levels of mice with dampness obstructing spleen-stomach, and enhanced the immune function of the mice. Studies by Yinghui He et al. (He et al., 2006) and Shaobo Zong et al. (Zong et al., 2015) discovered that HXZQC had therapeutic effects in mice with *Bacillus dysenteriae* and *Salmonella typhimurium*-induced diarrhea (BSD mice), mice with bacterial enteritis, and model rats with diarrhea-predominant irritable bowel syndrome (IBS). Clinical symptoms were significantly improved, which might be due to effects on the balance of CD4^+^ and CD8^+^ T lymphocytes, and reduction of interleukin-2 (IL-2), interleukin-10 (IL-10), interleukin-1β (IL-1β), and tumor necrosis factor alpha (TNF-α) levels. The results suggested that HXZQC, *via* immune-regulation and anti-inflammatory activity, could have therapeutic effects against many gastrointestinal disorders. Research by Hefei Huang et al. (Huang et al., 2016) showed that HXZQC extracts had a positive regulatory effect on intestinal dysfunction, and had therapeutic efficacy in model rats with diarrhea-predominant IBS. Efficacy was mediated by improving serum NO levels and reducing the concentrations of 5-hydroxytryptamine (5-HT), plasma motilin, and colonic somatostatin.

### Clinical Research on HXZQC

Modern clinical studies have shown that HXZQC has therapeutic effects against viral diseases, such as gastrointestinal-type cold, influenza, upper respiratory infection (URI), and viral enteritis (see **Table 3**). The Diagnosis and Treatment Scheme of Severe Acute Respiratory Syndrome (SARS) (Version 2004) (Chinese Medical Association and China Association of Chinese Medicine, 2004) recommended that HXZQC could be used for advanced stage pulmonary closure.

Research by Shuping Ma et al. showed that, compared with the control group (Ribavirin, interferon), HXZQC + Western medicines (Ribavirin, interferon) had a significant effect on antidiarrheal time and shortened the total time course in the treatment of children with rotavirus enteritis (*p* < 0.05) (Ma and Wang, 2012). Xiaoping Han et al. (Han, 2016) randomized 78 influenza patients into control and observation groups. Patients in the control group were given oral oseltamivir phosphate, while patients in the observation group received HXZQC in addition to oseltamivir phosphate. Compared with those in the control group, patients in the observation group had faster relief of fever symptoms, muscle aches, and fatigue (*p* < 0.05). The total response rate in the observation group was 97.44%, which was higher than the 82.05% of the control group (*p* < 0.05). The results showed that HXZQC enhanced the efficacy of oseltamivir phosphate in the treatment of influenza. Xingzhou Wu (Wu, 2010) randomized 90 cold dampness patients into two groups: the treatment group received HXZQP, while the control group received Ribavirin injection + Compound paracetamol and amantadine hydrochloride capsules. The total response rate in the treatment group was 88.9% compared to 77.1% in the control group (*p* < 0.05). Patients in the treatment group exhibited greater improvements in aversion to cold, fever, nasal congestion, running nose, spontaneous sweating, headache, cough and spitting, fatigue and weakness, body ache, and other cold symptoms compared to the control group. Hongjie Zhao et al. studied the efficacy and safety of HXZQC in the treatment of gastrointestinal-type cold by systematic evaluation. A total of 680 patients in eight randomized controlled trials (RCTs) were included in the research, and the results showed that the group receiving HXZQC had a significantly better clinical response than the group using Western medicines alone. The effects of HXZQC were superior to Western medicines in improving single symptoms (such as aversion to cold, fever, bowel sound and diarrhea) (Zhao et al., 2017). Dandan Yu et al. conducted a meta-analysis of 44 studies, including a total of 4,153 patients with acute gastroenteritis. The results showed that treatment with HXZQP + conventional therapy or norfloxacin tablets was superior to a single Western medicine in terms of total response rate and improvement of clinical symptoms (Yu et al., 2019).

### Usage and Dosage of HXZQC

Oral administration, four capsules, twice a day.

### Adverse Reactions of HXZQC

Potential drug eruption, purpura, shock, asthma, intestinal obstruction, upper gastrointestinal hemorrhage, hypoglycemia of childhood, infantile convulsions.

### HXZQC Precautions

1) Nourishing traditional Chinese medicines should not be taken during the period of medication. 2) It is advisable that patients are on a light diet during the period of medication.

## Relevant Information on the Clinical Application of LHQWC

### Recommended Therapeutic Regimens

LHQWC has been recommended in 19 therapeutic regimens for treatment of COVID-19 in China (see detailed information in **Tables 1** and **2**).

### Ingredients of LHQWC

*Forsythia suspensa* (Thunb.) Vahl (Lianqiao), *Lonicera japonica* Thunb. (Jinyinhua), *Ephedra equisetina* Bunge (Zhimahuang), *Prunus armeniaca* L. (Chaoxingren), Gypsum fibrosum (Shigao), *Isatis tinctoria* L. (Banlangen), *Dryopteris crassirhizoma* Nakai (Guanzhong), *Houttuynia cordata* Thunb. (Yuxingcao), *Pogostemon cablin* (Blanco) Benth. (Guanghuoxiang), *Rheum palmatum* L. (Dahuang), *Rhodiola rosea* L. (Hongjingtian), Mentholum (Bohenao), and *Glycyrrhiza uralensis* Fisch. ex DC. (Gancao). Basic information on LHQWC is provided in the **Supplementary Table**.

### Indications for the Treatment of COVID-19 With LHQWC

LHQWC is used during the clinical observation period of COVID-19, and wind-heat invading lung in early stage of the disease (mild case). The indicative symptoms are fever, mild aversion to cold, cough, weakness, headache and body pain, sore throat, and constipation.

### Progress of Pharmacological Research on LHQWC

Modern pharmacological studies have shown that LHQWC has antiviral, immune-regulatory, anti-inflammatory, and antioxidant properties, efficacy against lung injury, and other effects (see **Table 3**).

LHQWC significantly inhibited SARS-CoV-2 replication in Vero E6 cells and markedly reduced the production of pro-inflammatory cytokines (TNF-α, IL-6, CCL-2/MCP-1, and CXCL-10/IP-10) at the mRNA level. Furthermore, LHQWC treatment resulted in abnormal virion particle morphology in cells. LHQWC significantly inhibits SARS-CoV-2 replication, affects virus morphology and exerts anti-inflammatory activity *in vitro* (Li R. F. et al., 2020).

*In vitro* experiments have shown significant antiviral activity against SARS-CoV, AIV H7N9, dual H1N1/H3N2, together with inhibition of Middle East Respiratory Syndrome (MERS)-CoV activity to a certain degree (Yao et al., 2020). Yuewen Ding et al. used MTT and plaque reduction assays to show that LHQWC inhibited proliferation of multiple strains of influenza virus, and reduced virus titer and levels of inflammatory cytokines in the lungs of infected mice. The results indicated that LHQWC acted as a broad-spectrum antiviral and, in particular, regulated the immune response to viral infection (Ding et al., 2017). Qi Li et al. discovered that LHQWC not only reversed LPS-stimulated expression of macrophage chemotactic factor-1 (MCP-1) by macrophages, but also significantly improved pulmonary edema in a mouse model of acute lung injury. Inhibition of expression and secretion of MCP-1 in lung tissues of model mice was accompanied by reduced infiltration of mononuclear macrophages and reduction of inflammatory injury (Li et al., 2019). Fen Ping et al. studied the effects of LHQWC on rats with oxidative lung injury caused by fine particulate matters (PM 2.5). The results showed that LHQWC significantly reduced lactate dehydrogenase (LDH) and malondialdehyde (MDA) serum levels in rats with lung injury, increased levels of glutathione peroxidase (GSH-Px), reduced pathological damage of lung tissues, and inhibited exudation of inflammatory cells into the alveolar cavity. Together, the data indicated that LHQWC protected against oxidative stress injury in the lungs of rats (Ping et al., 2016). Wenwen Cui et al. studied the impact of LHQWC in a mouse model of acute lung injury caused by intratracheal infusion of LPS. LHQWC alleviated the inflammatory response in lung tissues by downregulating the IKK/IκB/nuclear factor (NF)-κB signaling pathway, thus, protecting mice from acute lung injury (Cui et al., 2016). Siwen Tang et al. studied the effects of LHQWC intervention on pathological lung tissue injury in mice and expression of inflammatory cytokines caused by exposure to automobile exhaust. The results showed that LHQWC reduced protein and mRNA expression of inflammatory cytokines in lung tissue by reducing blood levels of inflammatory cytokines, thus, protecting against lung tissue injury caused by automobile exhaust (Tang et al., 2015).

### Clinical Research on LHQWC

Modern clinical studies have shown that LHQWC has therapeutic effects against viral diseases, such as COVID-19, SARS, MERS, influenza, and human infection with H7N9 avian influenza. It can also be used to treat URI, chronic obstructive pulmonary disease (COPD) and other conditions (see **Table 3**). LHQWC has been recommended in diagnosis and treatment schemes such as China’s SARS Diagnosis and Treatment Scheme (Version 2004) (Chinese Medical Association and China Association of Chinese Medicine, 2004), MERS Diagnosis and Treatment Scheme (Version 2015) (National Health and Family Planning Commission of People’s Republic of China, 2015), China’s Influenza Diagnosis and Treatment Scheme (Version 2019) (National Health Commission of the People's Republic of China and National Administration of Traditional Chinese Medicine, 2019), and Diagnosis and Treatment Scheme for Human Infection with H7N9 Avian Influenza (Version 1, 2017) (National Health and Family Planning Commission of People's Republic of China, 2017).

Ke Hu et al. conducted a prospective multicenter open-label randomized controlled trial on LHQWC capsule in confirmed cases of COVID-19. Patients (284) were randomized to receive usual treatment alone or in combination with LHQWC capsules (four capsules, thrice daily) for 14 days. The primary endpoint was the rate of symptom (fever, fatigue, coughing) recovery. The recovery rate was significantly higher in the combined treatment group compared with the control group (91.5% vs. 82.4%, *p* = 0.022). The median time to symptom recovery was markedly shorter in the combined treatment group (median: 7 vs. 10 days, *p* < 0.001). Time to recovery of fever (2 vs. 3 days), fatigue (3 vs. 6 days) and coughing (7 vs. 10 days) was also significantly shorter in the combined treatment group (all *p* < 0.001). The rate of improvement in chest computed tomographic manifestations (83.8% vs. 64.1%, *p* < 0.001) and clinical cure (78.9% vs. 66.2%, *p* = 0.017) were also higher in the combined treatment group. However, the two groups did not differ in the rate of conversion to severe cases or viral assay findings (p > 0.05). No serious adverse events were reported (Hu et al., 2020).

Ruibing Lyu et al. conducted clinical research on 63 patients receiving conventional therapy in combination with LHQWC (treatment group) and 38 patients receiving only conventional therapy (control group). Clinical data were collected 10 days after the treatment. A comparison between the two groups was performed in terms of disappearance rates of cardinal symptoms (fever, cough, and weakness), duration of fever and disappearance rates of other individual symptoms and signs. The disappearance rates of fever, cough, and weakness in the treatment group were 86.7%, 55.6%, and 82.5%, respectively, which were higher than those in the control group (67.7%, 30.6%, and 58.6%; *p* < 0.05). The median duration of fever was 6 days in patients in the treatment group and 7 days in the control group. There was no statistically significant difference between the groups (*p* = 0.171). The disappearance rates of short breath and moist crackles (68.2% and 56.0%) were higher than those in the control group (20.0% and 20.0%, *p* < 0.05). There were four cases of aggravation in the treatment group (6.4%) and six cases in the control group (15.8%), with no statistically significant difference (*p* > 0.05). There were no obvious adverse reactions in the treatment group (Lyu et al., 2020).

Dezhong Cheng et al. conducted a multi-center retrospective analysis of the therapeutic effect of LHQWC in 51 COVID-19 patients. The control group was treated with simple nutritional support, symptomatic treatment, antiviral therapy and antimicrobial therapy. The treatment group was combined with LHQWC (6 g/bag) on the basis of the control group, one bag each time, 3 times a day. The clinical data of patients treated for 7 days were collected. The results showed that combined application of LHQWC significantly improved fever, weakness, cough, shortness of breath, chest distress, anorexia, and other clinical symptoms of COVID-19. Improvements of the main symptoms and reduced incidence of the severe form suggested that LHQWC could be effective in the treatment of patients with COVID-19 (Cheng et al., 2020).

Ping Yu et al. conducted a study on the therapeutic effect of LHQWC combined with Abidole in the treatment of mild COVID-19. A total of 295 patients were randomly divided into two groups. The control group (n = 148) was treated with Abidole (0.2 g per day) orally, and the observation group (n = 147) was treated with LHQWC (6 g, thrice daily) combined with Abidole. The results showed that the total effective rate of the observation group was significantly higher than that of the control group (80.95% vs 64.86%), and the rate of severe illness was significantly lower than that of the control group (14.29% vs 23.65%). After 7 days of treatment, the scores for the main TCM syndromes (fever, fatigue, cough, dry throat, chest tightness) and the levels of C-reactive protein (CRP) and procalcitonin (PCT) in the observation group were significantly lower than those in the control group (*p* < 0.05), while white blood cells (WBC) and lymphocyte (LYM) were significantly higher than those in the control group. The effective rate of chest computerized tomography (CT) in the observation group was 69.39%, which was higher than that in the control group (62.84%), but the difference was not statistically significant (*p* > 0.05). There were no serious drug-related adverse reactions in either group. The results show that LHQWC combined with Abidole can effectively relieve clinical symptoms in patients with mild COVID-19, regulate the expression of related inflammatory factors, improve the curative effect and reduce the rate of severe illness (Yu P. et al., 2020).

Lili Liu et al. conducted a retrospective analysis of the therapeutic effect of LHQWC in 32 COVID-19 patients. The patients were divided into two groups: Group A + L, in which 18 patients received Abidole (0.2 g, thrice daily) combined with LHQWC; and Group L, in which 14 patients received LHQWC alone. During treatment there was one critical case in each group. Abnormal liver function was observed in 14 cases (77.78%) in Group A + L and 8 cases (57.14%) in Group L. Antibiotic treatment was applied in 17 cases (94.44%) in Group A + L and 13 cases (92.86%) in Group L. Glucocorticoid use was reported in 10 cases (55.56%) in Group A + L and 9 cases (64.29%) in Group L. Compared with Group L, significantly faster recovery of temperature (t = −2.471, *p* = 0.019), recovery of respiratory symptoms (t = −2.918, *p* = 0.007), chest CT inflammation absorption (t = −2.937, *p* = 0.006), time until two consecutive negative virus nucleic acid tests (t = −2.930, *p* = 0.006), and shorter hospital stay (t = −2.785, *p* = 0.009) were observed in Group A + L. Abidor combined with LHQWC can be used to treat COVID-19, with good tolerance, to shorten the course of treatment (Liu et al., 2020).

Zhongping Duan et al. conducted a random, double-blind, and positive-drug parallel control clinical trial on the efficiency and safety of LHQWC against H1N1. It was found that LHQWC reduced disease severity and the duration of symptoms. The drug was also well tolerated, indicating that LHQWC might become an alternative therapeutic measure against H1N1 viral infection (Duan et al., 2011). Pan Zhao et al. found by meta-analysis that LHQWC improved cough, sore throat, body ache, and other symptoms of patients infected with H1N1 virus, reduced the duration of fever, and was more effective than oseltamivir (Zhao P. et al., 2014). Shiheng Wang et al. conducted a systematic review of the literature on efficacy and safety of LHQWC in treating viral flu. The results showed that LHQWC gave a better total response rate, symptom improvement rate and body temperature recovery rate than the control group in treating viral flu, but consideration of its safety was important (Wang et al., 2019). Li Tiehui et al. compared the clinical therapeutic effect of LHQWC and vitamin C Yinqiao Tablets in patients with URI, and found that LHQWC significantly improved nasal congestion, fever, headache, sore throat, weakness, aches in the limbs, intolerance of cold, and other clinical symptoms. LHQWC had high efficacy and safety, and was therefore worthy of promotion (Li, 2019). Dong Liang et al. conducted clinical research on 100 patients with COPD, and discovered that LHQWC improved conditions in patients with acute exacerbation of chronic obstructive pulmonary disease (AECOPD), especially those in the high risk subgroup. The mode of action might be related to its ability to reduce release of inflammatory mediators (Dong et al., 2014).

### Usage and Dosage of LHQWC:

Oral administration, four capsules, 3 times a day.

### Adverse Reactions of LHQWC

Possible nausea, vomiting, diarrhea, stomach discomfort, heartburn, poor appetite, and other gastrointestinal adverse reactions; there might be abnormal liver function, palpitations or rash, and other side effects occasionally.

### LHQWC Precautions

1) Pregnant and lactating women should use with caution. 2) It contains ephedrae herba (Mahuang), so should be used with caution by athletes and patients with high blood pressure and heart disease. 3) Those with previous history of liver disease or with abnormal liver function should use with caution. 4) It contains rheum, so the dose should be reduced appropriate in subjects who experience increased stool frequency and shapeless stools after administration. 5) Nourishing traditional Chinese medicine should not be taken at the same time.

## Relevant Information on the Clinical Application of JHQGG

### Recommended Therapeutic Regimens

JHQGG has been recommended in 13 therapeutic regimens of COVID-19 in China (see detailed information in **Tables 1** and **2**).

### Ingredients of JHQGG

*Forsythia suspensa* (Thunb.) Vahl (Lianqiao), *Lonicera japonica* Thunb. (Jinyinhua), *Ephedra equisetina* Bunge (Zhimahuang), *Prunus armeniaca* L. (Chaoxingren), Gypsum Fibrosum (Shigao), *Scutellaria baicalensis* Georgi (Huangqin), *Fritillaria thunbergii* Miq. (Zhebeimu), *Anemarrhena asphodeloides* Bunge (Zhimu), *Arctium lappa* L. (Niubangzi), *Artemisia annua* L. (Qinghao), *Mentha canadensis* L. (Bohe), and *Glycyrrhiza uralensis* Fisch. ex DC. (Gancao). Basic information on JHQGG is provided in the **Supplementary Table**.

### Indications for the Treatment of COVID-19 With JHQGG

JHQGG is used to treat the syndrome of wind-heat invading lung during the clinical observation period of COVID-19 and the early stage of the disease (mild case). Indicative symptoms are fever, mild aversion to cold, weakness, cough, headache and body pain, and sore throat.

### Progress of Pharmacological Research on JHQGG

Modern pharmacological studies have found that JHQGG has antiviral, immune-regulatory, and anti-inflammatory effects (see **Table 3**).

Jianping Qi et al. showed that JHQGG significantly decreased the average levels of C-reactive protein (CRP) and IFN-γ in serum of influenza patients. Patients exhibited decreased inflammatory response and improved immune function after treatment, which might be due to the antiviral activity of the main ingredients, such as Lonicerae japonicae flos, Scutellariae radix, Forsythiae fructus, and Artemisiae annuae herba (Qi et al., 2016).

### Clinical Research on JHQGG

Modern clinical studies have shown that JHQGG has therapeutic efficacy against viral diseases (see **Table 3**). JHQGG has been recommended in China’s Influenza Diagnosis and Treatment Scheme (Version 2019).

COVID-19 outpatients (123) were randomly divided into a treatment group (JHQGG two bags per time, 3 times a day, combined with routine treatment for 5 days, n = 82) and a control group (only routine treatment, n = 41). The addition of JHQGG significantly alleviated fever, cough, fatigue, sputum and anxiety, and the hospitalization rate tended to be lower than in the control group (Duan et al., 2020). In treatment of H1N1, use of JHQGG alone or in combination with oseltamivir effectively shortened fever duration. The duration of fever in patients treated with oseltamivir in combination with JHQGG was significantly shorter (19%) than in those treated with oseltamivir alone, suggesting that JHQGG could serve as an alternative therapeutic measure against H1N1 (Wang C. et al., 2011). Jianping Qi observed 174 cases of influenza patients and found that JHQGG significantly reduced serum levels of cytokines and improved immune function (Qi et al., 2016). A double-blind, randomized and controlled study on JHQGG in treating influenza syndrome of wind-heat invading lung by Guoqin Li et al. showed that it was effective and safe (Li et al., 2013).

### Usage and Dosage of JHQGG

Taken after dissolving in boiled water, one bag, 3 times a day.

### Adverse Reactions of JHQGG

Potential for nausea, vomiting, diarrhea, stomach discomfort, heartburn, poor appetite and other gastrointestinal adverse reactions; there might be abnormal liver function, palpitations, or rash occasionally.

### JHQGG Precautions

1) Those with deficiency-cold in spleen and stomach should use with caution. 2) It contains ephedrae herba (Mahuang), so should be used with caution by athletes and patients with high blood pressure and heart disease. 3) Those with previous history of liver disease or with abnormal liver function should use with caution. 4) Nourishing traditional Chinese medicine should not be taken at the same time.

## Relevant Information on the Clinical Application of SFJDC

### Recommended Therapeutic Regimens

SFJDC has been recommended in 15 therapeutic regimens of COVID-19 in China (see detailed information in **Tables 1** and **2**).

### Ingredients of SFJDC

Reynoutria japonica Houtt. (Huzhang), Forsythia suspensa (Thunb.) Vahl (Lianqiao), Isatis tinctoria L. (Banlangen), Bupleurum chinense DC. (Chaihu), Patrinia scabiosifolia Link (Baijiangcao), Verbena officinalis L. (Mabiancao), Phragmites australis (Cav.) Trin. ex Steud. (Lugen), and Glycyrrhiza uralensis Fisch. ex DC. (Gancao). Basic information on SFJDC is provided in the **Supplementary Table**.

### Indications for the Treatment of COVID-19 With SFJDC

SFJDC is used to treat external wind-heat syndrome during the clinical observation period of COVID-19 and the early stage of the disease (mild case). Indicative symptoms are fever, aversion to cold, cough with yellow phlegm, weakness, and sore throat.

### Progress of Pharmacological Research on SFJDC

Modern pharmacological studies have found that SFJDC has antiviral, antibacterial, and anti-inflammatory properties and protects against lung injury (see **Table 3**).

Yanyan Bao et al. evaluated the broad-spectrum antiviral activity of SFJDC by cytopathic effect (CPE) inhibition. A total of eight viruses, including H1N1, herpes simplex (HSV), respiratory syncytial virus, adenovirus (ADV) and Coxsackie virus, were evaluated. SFJDC had significant *in vitro* broad-spectrum antiviral activity and the best inhibitory effect was against parainfluenza virus (PIV). Similar results were obtained from *in vivo* experiments (Qiu et al., 2014; Bao et al., 2019). Ying Liu et al. used H1N1 FM1 and PR8 strains to induce nasal drip infection in an immunocompromised mouse pneumonia model. Therapeutic and preventive effects of SFJDC were observed against H1N1 infection *in vivo*. The results showed that SFJDC influenced the immune function of the mice, improved pneumonia symptoms caused by influenza virus, reduced the lung index of mice infected with H1N1, significantly reduced mortality, and had good therapeutic efficacy (Liu et al., 2010). Research by Weiwei Lv et al. found that SFJDC had inhibitory activity against multiple viruses and bacteria. Its antiviral activity was inferior to that of Ribavirin, but its cytotoxicity was lower. Both antiviral activity and antibacterial action were superior to those of Qingkailing granules (QKLG) (Lv et al., 2013). Li Ma et al. used a mouse pneumonia model induced by *Streptococcus pneumoniae* to study the anti-inflammatory mechanism of SFJDC. They discovered that it reduced white blood cell (WBC) count, reduced serum levels of the transcription factor nuclear factor kappa B (NF-κB), MCP-1, inflammatory mediator BK and COX-2, thus, having a therapeutic effect in the model (Ma et al., 2018). Further studies found that SFJDC had a significant immune regulatory function, reducing levels of B lymphocytes, CD8^+^ cells, interleukin-1α (IL-1α), IL-1β, IL-2, IgM, and IgG to improve lung function in mice with pneumonia. SFJDC increased the CD4^+^/CD8^+^ ratio and number of natural killer (NK) cells, thus, having a therapeutic effect in the pneumonia model (Ma et al., 2019a; Ma et al., 2019b). Zhengang Tao et al. observed a protective function of SFJDC against endotoxin LPS-induced rat lung injury. Their results showed that SFJDC inhibited the LPS-induced inflammatory response, and reduced LPS-induced lung injury. Its mechanism of action might be inhibition of the MAPK (mitogen-activated protein kinase)/NF-κB signaling pathway and downregulation of NF-κB mRNA expression (Tao et al., 2014). Yanmei Li et al. used a *Pseudomonas aeruginosa* (PAK)-induced KM mouse acute lung injury model to explore the mode of action of SFJDC in treatment of acute lung injury. They found that SFJDC significantly alleviated lung injury in the model and its mode of action might be related to the ERK signaling pathway (Li et al., 2017).

### Clinical Research on SFJDC

SFJDC comes from “Detoxification Powder,” and is mainly used to treat fever, parotitis, amygdalitis, plague, and other diseases. Recent studies have shown that SFJDC has good clinical efficacy against viral diseases (such as MERS, influenza, human infection with H7N9 avian influenza) and respiratory diseases (such as acute URI, AECOPD, pneumonia, etc.) (see **Table 3**). SFJDC has been recommended in diagnosis and treatment schemes such as MERS Diagnosis and Treatment Scheme (Version 2015), China’s Influenza Diagnosis and Treatment Scheme (Version 2019), and Diagnosis and Treatment Scheme for Human Infection with H7N9 Avian Influenza (Version 1, 2017).

Lei Wang et al. conducted a retrospective analysis of 87 patients with acute URI + fever, and found that patients treated with SFJDC had a significantly higher total response rate than those in the control group. SFJDC effectively improved respiratory symptoms in patients with acute URI + fever (Wang and Qiu, 2018). In the treatment of community acquired pneumonia (CAP), application of SFJDC shortened recovery time, reduced the duration of fever and reduced the levels of procalcitonin (PCT), CRP, WBC, and other indicators, effectively shortening the course of treatment (Wang, 2016). Hongxia Yu et al. evaluated the impact of SFJDC on inflammation-associated cytokines in patients with AECOPD. The results showed that SFJDC significantly reduced the levels of interleukin-8 (IL-8), TNF-α, CRP, and PCT, and had significant therapeutic efficacy against AECOPD (Yu H. X. et al., 2020). Tiling Wang et al. added SFJDC treatment to conventional treatment in 60 mild and moderate AECOPD patients and compared with 60 patients receiving conventional treatment as the control group. After 1 week, the treatment group had significantly higher arterial blood gas PaO_2_ than the control group, without any adverse reactions (Wang, 2015). Research showed that treatment of bacterial acute bronchitis and pneumonia with a combination of antibacterial drugs and SFJDC significantly shortened recovery of body temperature, duration of cough and the course of treatment compared with antibacterial drug alone. Chunlan Wang et al. observed that combined use of SFJDC and antibiotics significantly improved body temperature, blood sugar, ALT (glutamate transaminase), AST (aspartate aminotransferase) and other indicators compared with the control group, and patients had a higher oxygen index than the control group. The results suggested that SFJDC had a significant protective function against lung injury, and the mechanism might be related to inhibition of inflammatory response by SFJDC (Wang et al., 2014).

### Usage and Dosage of SFJDC

Oral administration, four capsules, 3 times a day.

### Adverse Reactions of SFJDC

Occasional nausea.

### SFJDC Precautions

(1) Use is forbidden in those with allergic constitution or who are allergic to the drug. (2) Use is forbidden in those with deficiency-cold in spleen and stomach.

## Relevant Information on the Clinical Application of TRQI

### Recommended Therapeutic Regimens

TRQI has been recommended in 12 therapeutic regimens of COVID-19 in China (see detailed information in **Tables 1** and **2**).

### Ingredients of TRQI

*Scutellaria baicalensis* Georgi (Huangqin), Ursi fellis pulvis (Xiongdanfen), *Forsythia suspensa* (Thunb.) Vahl (Lianqiao), and *Lonicera japonica* Thunb. (Jinyinhua). Basic information on TRQI is provided in the **Supplementary Table**.

### Indications for the Treatment of COVID-19 With TRQI

TRQI is used for syndromes of epidemic toxin lung closure and phlegm-heat lung obstruction in the progressive stage of COVID-19 (critical case). Indicative symptoms are fever, cough, cough with difficulty in expectoration, chest distress, and shortness of breath.

### Progress of Pharmacological Research on TRQI

Modern pharmacological studies have shown that TRQI is effective against influenza virus, destroys bacterial biofilm, inhibits airway inflammation, and improves lung injury (see **Table 3**).

Research by Jinsu Zheng et al. discovered that TRQI improved pathological injury of lung tissues in mice infected with influenza virus, and had significant antiviral activity in influenza virus infected mice. The antiviral activity of TRQI might be due to its inhibition of cellular proliferation and enhancement of immunity (Zheng and Gu, 2009). Weifeng Yang et al. discovered that TRQI could destroy methicillin-resistant *Staphylococcus aureus* (MRSA) biofilm and induce its death. When combined with vancomycin or linezolid below the minimal inhibitory concentration (MIC) concentration, synergistic anti-biofilm activity was observed that was significantly higher than when using TRQI alone (Yang et al., 2018). Research by Yi Wang et al. showed that the efficacy of TRQI in the treatment of acute pneumonia was mediated by destruction of bacterial biofilm, which is different to the mechanism of penicillin (Wang Y. et al., 2011). Wei Liu et al. discovered that TRQI might treat airway mucus hypersecretion by regulating the interleukin-17 (IL-17) signaling pathway and its downstream protein MUC5AC. An *in vivo* experiment showed that TRQI could significantly inhibit excessive secretion of LPS-stimulated MUC5AC and expression of TNF-α, interleukin-6 (IL-6), IL-8, and IL-17A in terms of protein and mRNA levels (Liu W. et al., 2019). Animal experiments conducted by Wei Liu et al. showed that TRQI inhibited airway inflammation caused by LPS through the MAPK/NF-κB pathway, and showed a dose-dependent effect (Liu et al., 2016). Li Wen et al. found that TRQI improved signs and symptoms in AECOPD patients, which might be mediated by reduction of serum IL-8 and neutrophil elastase (NE) levels, and improved airway inflammation and mucus hypersecretion (Li et al., 2010). Research by Li Pengtao et al. discovered that TRQI improved blood flow in capillaries of the alveolar walls while repressing the LPS-induced inflammatory cascade, which was the pharmacological basis for its effective alleviation of acute lung injury and prevention of decreased arterial partial oxygen pressure (Li et al., 2005).

### Clinical Research on TRQI

Modern clinical studies have shown that TRQI has therapeutic efficacy against infectious diseases, such as viral pneumonia, MERS, human infection with H7N9 avian influenza, acute bronchitis, acute attack of chronic bronchitis, CAP, tuberculosis accompanied by infection, and AECOPD (see **Table 3**). TRQI has been recommended in MERS Diagnosis and Treatment Scheme (Version 2015) and Diagnosis and Treatment Scheme for Human Infection with H7N9 Avian Influenza (Version 1, 2017).

A systematic evaluation of eight published randomized and controlled trials that included a total of 590 adult patients with viral pneumonia found that TRQI had advantages in terms of response rate, faster change of chest radiography, average length of stay, and other aspects (Pan, 2016). Research results from Jinzhi Liang et al. showed that there was no statistically significant difference in the clinical effect of combined TRQI and Ribavirin or TRQI alone in the treatment of hand-foot-and-mouth disease. Both treatments were superior to that of Ribavirin alone (Liang et al., 2013). Research by Wang Pei et al. showed that potential benefits of TRQI in the treatment of acute bronchitis included improved response rate, and reduced fever, cough, crackles, and X-ray shadow absorption (Wang et al., 2016). Research results of Lini Gao et al. showed that combined use of TRQI and Western medicines was more effective than Western medicines alone in the treatment of acute bronchitis and gave superior improvement of clinical symptoms (Gao et al., 2019). Hongli Jiang et al. showed by systematic evaluation that administration of TRQI to treat CAP on the basis of antibiotics and symptomatic treatment significantly improved clinical symptoms. Cough with expectoration was improved, the duration of fever was shortened and recovery of chest radiography and hemogram were promoted without significant adverse reactions (Jiang et al., 2009). Lian Xiong et al. showed by systematic evaluation that TRQI might have the same overall effect as some antibacterial drugs in treatment of patients with tuberculosis accompanied by lung infection, but improved efficacy was observed in combination with antibacterial drugs. This might be due to the bacteriostatic effects of TRQI and elimination of inflammatory mediators (Lian et al., 2018). A total of 14 trials and 954 patients were included in a study by Yunqing Zhong, and the results showed that combined use of TRQI and antibacterial drugs improved the clinical effects and lung function in AECOPD patients, reduced pCO_2_, and shortened the length of stay without serious adverse reactions (Zhong et al., 2010).

### Usage and Dosage of TRQI

20 ml once for adults, 40 ml once for severe patients, with addition of 250–500 ml 5% glucose or 0.9% sodium chloride; intravenous drip at less than 60 drops per min, once a day.

### Adverse Reactions of TRQI

1) Some patients may have dizziness, chest distress, nausea, vomiting, and diarrhea. 2) Flushing, rash or itching and other allergic reactions occasionally. 3) Rarely, palpitations, chill and difficulty breathing. 4) Extremely rarely, allergic shock. 5) Other adverse reactions: dry mouth, fever, periorbital facial edema, discomfort at infusion site.

### TRQI Precautions

1) Use is forbidden in those with liver and renal failure; 2) Use is forbidden in those with severe lung and heart disease accompanied by heart failure; 3) Use is forbidden in pregnant women and infants less than 24 months; 4) It should be used alone and must not be mixed with other drugs; 5) Dilution ratio of the liquid shall be no lower than 1:10 (liquid: solvent) and the diluted liquid must be used within 4 h.

## Relevant Information on the Clinical Application of XYPI

### Recommended Therapeutic Regimens

XYPI has been recommended in 15 therapeutic regimens of COVID-19 in China (see detailed information in **Tables 1** and **2**).

### Ingredients of XYPI

Andrographolide total sulfonate. Basic information on XYPI is provided in the **Supplementary Table**.

### Indications for the Treatment of COVID-19 With XYPI

XYPI is used for syndrome of exuberance of internal heat toxin in progressive stage of COVID-19 (critical case). Indicative symptoms are fever, sore throat, cough with yellow phlegm and chest distress. It could also be used to treat viral infection combined with mild bacterial infection.

### Progress of Pharmacological Research on XYPI

The main ingredient of XYPI is andrographolide total sulfonate, which is antipyretic, anti-inflammatory, antiviral, antibacterial and immune-regulatory (see **Table 3**).

Yang Yu et al. conducted *in vivo* experiments with XYPI, and discovered that it could significantly protect mice infected with *Staphylococcus aureus* and *Streptococcus pneumoniae*, and significantly inhibited citric acid-induced cough frequency in guinea pigs (Yu et al., 2009). Using *in vitro* experiments, Lu Wang et al. studied the inhibitory effect of XYPI on inflammatory factors released by LPS-stimulated mouse mononuclear macrophages. The results showed that XYPI significantly inhibited the release of inflammatory factors such as TNF-α and IL-6 (Wang et al., 2008). Yinglan Nie et al. explored the mode of action of XYPI in the treatment of acute lung injury by observing its effect on cytokine content in bronchoalveolar lavage fluid (BALF) following LPS-induced acute lung injury. The results showed that XYPI could play an anti-inflammatory role by modulating the balance of pro-inflammatory/anti-inflammatory cytokines and prevent excess anti-inflammatory responses during the course of acute lung injury (Nie et al., 2012). Qi Liu et al. observed antiviral activity of XYPI against human rhinovirus-induced mouse infections. XYPI significantly reduced the virus titer in trachea-lung tissue homogenate of infected mice, effectively inhibiting proliferation of human rhinovirus in mice. Respiratory lesions were alleviated in tested animals, survival rate was improved, there were few adverse reactions, and it was an efficient and safe drug against human rhinovirus infection. Its specific mode of action, however, was unclear (Liu, 2015).

### Clinical Research on XYPI

XYPI is a broad-spectrum antiviral Chinese patent medicine that is widely used to treat acute URI, viral pneumonia and pulmonary infection in clinical practice with good efficacy. Recent studies have discovered that it can also inhibit some viruses and bacteria, and could be used to treat influenza, human infection with H7N9 avian influenza, capillary bronchitis and other diseases (see **Table 3**). XYPI has been recommended in China’s Diagnosis and Treatment Scheme for Human Infection with H7N9 Avian Influenza (Version 2017).

Xiuping Yin et al. used the association rule method to analyze drug combinations, including XYPI in patients with pulmonary infection. The results showed that it could play a role as alternative or as a supplement to antibiotics in the treatment of pulmonary infection, but the safety and rationality of its use in drug combinations required further study in clinical practice (Yin et al., 2015). Guangming Li et al. conducted a retrospective analysis of 92 patients with viral pneumonia and found that XYPI was more effective than Ribavirin, providing significant improvement of symptoms (Li, 2015). Ruihan Qi et al. analyzed the therapeutic effect of XYPI in the treatment of viral pneumonia by systematic evaluation and found that it was more effective than Ribavirin. XYPI increased the cure rate, improved signs and symptoms, and reduced the incidence of adverse reactions (Qi et al., 2018). Lili Zhang et al. used XYPI in combination with Western medicine to treat severe pneumonia of the elderly in clinical practice. The results showed that it significantly shortened the course of disease, improved treatment efficiency, reduced the incidence of antibiotic resistance, reduced occurrence of double infection, improved the prognosis, and reduced mortality (Zhang and Wang, 2015). Zhixu Yang et al. observed the clinical effect of XYPI in treating the syndrome of phlegm-heat obstructing lung of severe pneumonia from the perspective of traditional Chinese medicine. The results showed significant improvements that included reduced fever, reduced numbers of leukocytes, improved oxygen index, lower clinical pulmonary infection score (CPIS), and reduced pulmonary inflammation. It also shortened the duration of mechanical ventilation and length of stay in ICU, and improved the clinical effect (Yang et al., 2014). In addition, XYPI has also shown significant efficacy in the treatment of URI. Xiaowen Liu et al. conducted a retrospective analysis of 660 patients with acute URI and found that the total response rate in the XYPI treatment group was significantly higher than that of the control group. The difference was statistically significant (Liu and Li, 2015).

### Usage and Dosage of XYPI

1) Intramuscular injection. Adults: 50-100 mg, 2 or 3 times a day. 2) Intravenous drip. Adults: 250–500 mg a day, diluted with 0.9% sodium chloride or 5% glucose.

### Adverse Reactions of XYPI

The main adverse reactions are allergic reaction, damage to the skin, damage to the digestive system, damage to the respiratory system, and general damage to the cardiovascular system. These are manifested by rash, itching, shivering, facial blushing, fever, cyanosis, difficulty breathing, nausea, vomiting, palpitations, chest distress, and allergic shock.

### XYPI Precautions

1) Use is forbidden in pregnant women and children under 1 year of age. Use with caution in the elderly above 75 years of age. 2) Use is forbidden in those with a history of allergic or severe adverse reactions to this drug or preparations containing andrographolide total sulfonate. 3) Enhanced monitoring is recommended in patients using XYPI for the first time; pay close attention to reactions during administration, especially if discovering abnormalities within 30 min of administration. Stop administration immediately and take active rescue measures. 4) When used in combination with other injections, XYPI should be administered first. Other injections can be infused after flushing or replacing the infusion tube.

## Relevant Information on the Clinical Application of XBJI

### Recommended Therapeutic Regimens

XBJI has been recommended in 20 therapeutic regimens of COVID-19 in China (see detailed information in **Tables 1** and **2**).

### Ingredients of XBJI

*Carthamus tinctorius* L. (Honghua), *Paeonia lactiflora* Pall. (Chishao), *Conioselinum anthriscoides* ‘Chuanxiong’ (Chuanxiong), *Salvia miltiorrhiza* Bunge (Danshen), and *Angelica sinensis* (Oliv.) Diels (Danggui). Basic information on XBJI is provided in the **Supplementary Table**.

### Indications for the Treatment of COVID-19 With XBJI

XBJI is used for syndrome of blood-stasis and toxins in the progressive stage of COVID-19 (critical case). Indicative symptoms are fever, dyspnea and tachypnea, palpitations, and dysphoria. It could also be used for treatment of infection-induced systemic inflammatory response syndrome and multiple-organ dysfunction syndrome in the stage of impaired organ function.

### Progress of Pharmacological Research on XBJI

Modern pharmacological studies have shown that XBJI is anti-inflammatory, antioxidant, immune-regulatory, and protects against acute lung injury (see **Table 3**).

Tiantian Li et al. found that in mice with MRSA-induced sepsis, XBJI protected the infected mice by downregulating expression of inflammatory cytokines stimulated by Pam3CSK4, MAPK, PI3K (phosphatidylinositol 3 kinase)/Akt and other pathways, thus, inhibiting the inflammatory response (Li T. T. et al., 2020). Shuwen Zhang et al. and Xi Chen et al. found that XBJI significantly reduced TNF-α, IL-6, and IL-10 levels in mice with sepsis, prevented neutrophil infiltration of lung and kidney, modulated T helper cell (Th) 1/Th2, Th17, and Tregs balance, reduced inflammatory response, and improved survival rate in mice with infectious shock (Zhang et al., 2006; Chen et al., 2018). Mingwei Liu et al. studied rats with paraquat-induced acute lung injury and discovered that XBJI could enhance immunity, reduce expression of inflammatory factors, and protect against acute lung injury by blocking p-38 MAPK and NF-κB p65 pathways, (Liu et al., 2014). Research by Yin Teng et al. found that XBJI in combination with conventional treatment significantly reduced interleukin-1 (IL-1), IL-6, and TNF-α levels, improved CD4^+^/CD8^+^ T lymphocyte ratio and NK cell relative activity, reduced inflammatory response, and enhanced cellular immunity in patients with severe pneumonia (Teng et al., 2012). Research by Hui Jin et al. showed that XBJI significantly improved the activity of superoxide dismutase (SOD), reduced reactive oxygen species (ROS) levels and protected against oxidative damage in mice under high-temperature stimulation (Jin et al., 2018). Research by Luo Peng et al. showed that XBJI downregulated MDA levels, upregulated SOD levels, and alleviated LPS-induced acute lung injury in rats (Luo and Zhou, 2017). In a rat model of oleic acid or LPS-induced acute lung injury, XBJI reduced TNF-α levels, alleviated pulmonary tissue edema and inflammatory cell infiltration, and protected against lung injury (Zhang et al., 2014). Research by Yuexia Ma et al. showed that although XBJI had no direct antiviral effect in mice with H1N1 severe pneumonia; it alleviated lung injury and protected against death, which might be due to its regulation of inflammatory cytokine levels in the early stage (Ma et al., 2015).

### Clinical Research on XBJI

Modern clinical studies have shown that XBJI in combination with conventional treatment has therapeutic effects in relevant diseases, such as MERS, human infection with H7N9 avian influenza, CAP, severe pneumonia, systemic inflammatory response syndrome, COPD and sepsis (see **Table 3**). XBJI has been recommended in MERS Diagnosis and Treatment Scheme (Version 2015) and Diagnosis and Treatment Scheme for Human Infection with H7N9 Avian Influenza (Version 1, 2017).

Clinical research by Wen Long et al. randomly divided 60 severe COVID-19 patients into routine treatment (n = 20), XBJI 50 ml (n = 20), and XBJI 100 ml (n = 20) groups. On the basis of conventional treatment, XBJI (50 ml) was injected twice a day for 7 days in the XBJI 50 ml group, or 100 ml twice a day for 7 days in the XBJI 100 ml group. After treatment, the white blood cell count (WBC) and lymphocyte count (LYM) of the three groups increased, while CRP and ESR decreased. Compared with the routine treatment group, the WBC count in the XBJI 100 ml group after treatment significantly increased (×10^9^/L: 7.12 ± 0.55 vs. 5.67 ± 0.51, *p* < 0.05), and the levels of CRP and ESR in the XBJI 50 ml and 100 ml groups significantly decreased [CRP (mg/L): 32.3 ± 4.6, 28.0 ± 6.2 vs. 37.3 ± 5.9; ESR (mm/h): 45.9 ± 5.7, 40.5 ± 7.4 vs. 55.3 ± 6.6, all *p* < 0.05]. Compared with the XBJI 50 ml group, the increase of WBC, and the decrease of CRP and ESR were more significant in the XBJI 100 ml group [WBC (×10^9^/L): 7.12 ± 0.55 vs. 5.82 ± 0.49; CRP (mg/L): 28.0 ± 6.2 vs. 32.3 ± 4.6; ESR (mm/h): 40.5 ± 7.4 vs. 45.9 ± 5.7, all *p* < 0.05]. The APACHE II score of three groups decreased. In the XBJI 100 ml group, the APACHE II score after treatment was significantly lower than those in the routine treatment and XBJI 50 ml groups (12.3 ± 1.5 vs. 16.5 ± 1.6, 15.9 ± 1.4, both *p* < 0.05). After treatment, the 2019-nCoV nucleic acid test in the three groups partly turned negative: nine cases in the routine treatment group, eight cases in the XBJI 50 ml group and nine cases in the XBJI 100 ml group, with no significant differences (*p* > 0.05). The conditions of patients in the three groups were improved after treatment. Eight cases in the routine treatment group were transformed into common type and one case into critical type; nine cases and 12 cases in the XBJI 50 ml and 100 ml groups, respectively, were transformed into the common type. Patients in the XBJI 100 ml group improved more obviously than in the XBJI 50 ml and routine treatment groups (both *p* < 0.05). The XBJI injection can effectively improve the inflammatory markers and prognosis of severe COVID-19 patients (Wen et al., 2020).

Clinical research by Qi Fei et al. showed that, of 80 patients with severe pneumonia, those receiving a combination of XBJI and conventional treatment exhibited reduced levels of blood LDH, α1-acid glycoprotein (α1-AG) and α1-antitrypsin (α1-AT). Body temperature was reduced significantly and secretion of TNF-α, IL-6, IL-8, and other cytokines was inhibited. The total treatment efficiency was up to 80%, compared to 67.5% in the control group (Qi et al., 2011). An RCT study comprised of 33 centers and 710 patients conducted by Yuanlin Song et al. showed that XBJI in combination with conventional treatment significantly improved the primary endpoint, pneumonia severity index, in patients with severe CAP (the control group vs XBJI Group, 46.33% vs 60.78%, *p* < 0.001). There was also significantly reduced mortality in 28 days (24.65% vs 15.87%, *p* = 0.006), the duration of mechanical ventilation was shortened (11 vs 16.5 d, *p* = 0.012) and length of stay in ICU was reduced (12 vs 16 d, *p* = 0.004) (Song et al., 2019). Mingjin Zhu et al. conducted a meta-analysis of 12 studies with a total of 860 patients and showed that XBJI in combination with conventional treatment was superior to the treatment group in improving total response rate in patients with severe pneumonia. Infectious indicators (WBC, CRP, CPIS) and inflammatory cytokine (IL-6, IL-8, TNF-α) levels were reduced, and the average length of stay in hospital was reduced (Zhu et al., 2014). Wei Zhao et al. studied 56 patients with systemic inflammatory response syndrome (SIRS) and found that after 7 d treatment with XBJI in combination with conventional treatment, body temperature, WBC, and acute physiology and chronic health evaluation II (APACHE-II) score improved more significantly compared to the control group (*p* < 0.05). Expression of CD4^+^, CD4^+^/CD8^+^, CD14^+^/HLA-DR (human leukocyte antigen-DR) increased significantly, and the combination regulated the SIRS immune state and improved systemic status of the patients (Zhao W. et al., 2014). Clinical research found that XBJI in combination with conventional treatment lowered TNF-α, CRP, and other inflammatory indicators in AECOPD patients and had a certain therapeutic effect. In patients with accompanying SIRS, the combination significantly improved cough, expectoration, shortness of breath, and other clinical symptoms, and shortened hospital stay (Chen et al., 2011; Zhu et al., 2019). Meta-analysis by Chengyu Li et al. included sepsis patients from 16 RCTs (total 1,144 cases), and evidence of moderate intensity showed that XBJI in combination with conventional treatment effectively reduced the mortality rate of sepsis patients over 28 d (934/1144, *p* < 0.00001), APACHE-II score (792/1144, *p* < 0.00001) and body temperature (362/1144, *p* < 0.00001) (Li et al., 2018).

### Usage and Dosage of XBJI

Intravenous injection. 1) Systemic inflammatory response syndrome: 50 ml plus 100 ml 0.9% sodium chloride injection for intravenous drip, completed in 30–40 min, twice a day. Three times a day for severe patients. 2) Multiple-organ dysfunction syndrome: 100 ml plus 100 ml 0.9% sodium chloride injection for intravenous drip, completed in 30–40 min, twice a day. Three or four times a day for severe patients.

### Adverse Reactions of XBJI

Allergic reactions: skin flush, rash, itching, palpitations, cyanosis, laryngeal edema, allergic shock, etc. Cardiovascular system: palpitations, cyanosis, increase, or decrease of blood pressure, arrhythmia. Nervous system: dizziness, headache. Respiratory system: difficulty breathing, chest distress, labored breathing, shortness of breath, and cough. Digestive system: nausea, vomiting, stomach ache, diarrhea, and abnormal liver function. Others: facial edema, conjunctival congestion, abnormal tears, phlebitis, lumbago, backache, and local numbness.

### XBJI Precautions

1) Not for use in pregnant women and children under 14 (inclusive) years of age. 2) The product must not be mixed with others, and must be used with caution in combination with others. When used in combination with other drugs, 50 ml 0.9% sodium chloride injection must be used between doses. 3) Allergic history, family allergic history and patient history of medications should be queried before administration. 4) During administration, special attention should be given to the initial 30 min of intravenous drip. In case of abnormality, the drug should be discontinued immediately and symptomatic treatment administered. 5) Monitoring of administration should be enhanced in older patients and in patients receiving TCM injection for the first time.

## Relevant Information on the Clinical Application of SFI

### Recommended Therapeutic Regimens

SFI has been recommended in 19 therapeutic regimens of COVID-19 in China (see detailed information in **Tables 1** and **2**).

### Ingredients of SFI

*Panax ginseng* C.A.Mey. (Hongshen) and *Aconitum carmichaeli* Debeaux (Fuzi). Basic information on SFI is provided in the **Supplementary Table**.

### Indications for the Treatment of COVID-19 With SFI

SFI is used for deliverance due to sudden yang deficiency in the progressive stage of COVID-19 (critical case). Indicative symptoms are dyspnea, pale complexion, and severe symptoms are unconsciousness, drip sweat, and cold limbs.

### Progress of Pharmacological Research on SFI

Modern pharmacological studies have shown that SFI has functions, including anti-shock, and protection from lung injury (see **Table 3**).

Yuhang Ai et al. explored the effects and mechanism of SFI in an LPS-induced lung injury model in rats. The results indicated that SFI might protect the lung by reducing activation of NF-κB in lung tissue (Ai et al., 2006). Research by Xia Liu et al. found that SFI improved the inflammatory response of rat lung tissue in an LPS shock model by reducing expression of p65 and p50 mRNA and protein in lung tissue and serum TNF-α (Liu et al., 2019a). Li Lin et al. studied the impact of SFI on LPS acute lung injury in rats, and found that SFI significantly increased the wet/dry weight ratio (W/D) of lung tissue, neutrophil ratio in BALF, protein content, lung tissue MDA, and serum NO. It significantly alleviated injury in lung tissue, indicating that SFI had an important preventive and therapeutic effect on LPS-induced acute lung injury (Lin and Zhan, 2010). Xi Liu et al. used the LPS intravenous injection method to establish a septic shock model in rabbits. Administration of SFI significantly improved mean arterial pressure (MAP), reduced LPS, LDH, and AST serum levels, and significantly improved the morphology of heart, liver, and kidney. In addition, SFI increased levels of adenosine triphosphate (ATP) and taurine in the heart, while reducing the level of adenosine monophosphate (AMP) in the heart. The results showed that SFI had a significant protective effect against LPS-induced septic shock (Liu et al., 2019b).

### Clinical Research on SFI

SFI is composed of *Panax ginseng* C.A.Mey. and *Aconitum carmichaeli* Debeaux, and has properties that include enhancing cardiac function, increasing blood pressure, and protecting ischemic myocardium. It is widely used to rescue from shock (infectious or cardiogenic shock) caused by various reasons, cardiac failure, and arrhythmia in clinical practice. Recent studies have shown that SFI significantly protects against lung injury (see **Table 3**). SFI has been recommended in China’s SARS Diagnosis and Treatment Scheme (Version 2004), the MERS Diagnosis and Treatment Scheme (Version 2015), and the Diagnosis and Treatment Scheme for Human Infections with H7N9 Avian Influenza (Version 2017).

Qiu Z.L. et al. observed a therapeutic effect of SFI in patients with severe sepsis and an impact on the expression levels of serum IL-6 and IL-10. They found that SFI significantly lowered IL-6 levels in patients with severe sepsis and regulated the balance between pro- and anti-inflammatory factors, thus, improving the therapeutic effect (Qiu et al., 2012). Ning Zhang et al. randomized 160 patients with sepsis into an SFI treatment group and a conventional treatment group. By collecting post-treatment immunological parameters, they conducted a comparative analysis of the impact on immune function. The results showed that patients in the SFI treatment group had increased CD4^+^ and CD8^+^ T cell counts in peripheral blood and upregulated HLA-DR expression in monocytes. In addition, the SFI treatment group had a better response than the control group for duration of vasopressor administration and APACHE-II score. The results showed that SFI enhanced cellular immune function in patients with septic shock and might become an important adjunctive therapy for sepsis patients (Zhang et al., 2017). Another study found that SFI played an active role in the treatment of severe pneumonia in the elderly. Among 89 elderly patients with severe pneumonia, the SFI treatment group had significantly decreased levels of TNF-α, IL-6, and IL-8 7 days after administration, indicating that SFI effectively reduced inflammatory mediators, thus, playing an active therapeutic effect (Lv et al., 2017). Min Ma et al. conducted clinical research on 80 patients with traumatic acute lung injury, and found that SFI significantly improved respiratory rate, improved the oxygen index, and reduced levels of intracellular adhesion molecule 1 (ICAM-1), endothelin-1 (ET-1), and NO, thus, improving prognosis of these patients. This study provided a potential new therapy for traumatic acute lung injury (Ma et al., 2019). Jie Li et al. observed an impact of SFI intervention on duration of mechanical ventilation in patients with respiratory failure. The results showed that the total response rate in the SFI group was higher than that of the control group. SFI significantly improved serum prealbumin and high-sensitivity CRP levels in patients with respiratory failure and improved their oxygen index, thus, shortening the duration of mechanical ventilation (Li, 2013).

### Usage and Dosage of SFI

1) Intravenous drip: 20–100 ml, diluted in 250–500 ml of 5%–10% glucose injection. 2) Intravenous injection: 5–20 ml, diluted in 20 ml of 5%–10% glucose injection.

### Adverse Reactions of SFI

Dizziness, headache, shivering, fever, palpitations, chest distress, chest pain, difficulty breathing, nausea, retching, abdominal pain, rash, itching, rash or swelling, pain, and other discomfort in local infusion site.

### SFI Precautions

1) To be used with caution in pregnant women. 2) Avoid direct mixing with coenzyme A, VitK3, and aminophylline. 3) Prepared drug should be used within 4 h.

## Relevant Information on the Clinical Application of SMI

### Recommended Therapeutic Regimens

SMI has been recommended in 18 therapeutic regimens of COVID-19 in China (see detailed information in **Tables 1** and **2**).

### Ingredients of SMI

*Panax ginseng* C.A.Mey. (Hongshen) and *Ophiopogon japonicus* (Thunb.) Ker Gawl. (Maidong). Basic information on SMI is provided in the **Supplementary Table**.

### Indications for the Treatment of COVID-19 With SMI

SMI is used for the syndrome of deficiency of both qi and yin and deficiency of pulse in the progressive stage of COVID-19 (critical case). Indicative symptoms are weakness and shortness of breath, tachypnea, palpitations, dry mouth, sweating, and even dysphoria and cold limbs.

### Progress of Pharmacological Research on SMI

Modern pharmacological studies have shown that SMI has functions that include protection from inflammatory shock, protection of heart and lung function, and immunoregulation (see **Table 3**).

Y. Z. Zhang et al. observed that SMI had strong anti-shock and neuroprotective properties in LPS-induced shock, possibly due to inhibition of brain lipid peroxidation and improvement of SOD activity (Zhang Y. Z. et al., 2010). SMI suppressed apoptosis of lung tissue cells during pulmonary ischemia/reperfusion injury in rabbits, resulting in attenuation of pneumocyte injury by raising NO levels, lowering oxygen free radical levels, and decreasing lipid peroxidation (Lin et al., 2007). It has also been reported that SMI reduced expression of NF-κB and activity of inducible nitric oxide synthase (iNOS) in lung tissues of rats poisoned by paraquat, and significantly alleviated erythrocyte diapedesis in the alveolar space (Liu et al., 2009). Research by Shuhua Xu et al. showed that SMI improved cardiac function and significantly improved hemodynamics in rats with cardiac failure. In addition, it improved the oxygen supply to tissues and the capacity of the tissues to use oxygen, thus, improving oxygen metabolism (Xu and Liu, 2010). SMI also inhibited expression of ICAM-1 and vascular cell adhesion molecule 1 (VCAM-1) to alleviate inflammatory infiltration following ischemia/reperfusion, and alleviated myocardial ischemia/reperfusion injury caused by multiple inflammatory responses (Liu et al., 2015). It also inhibited generation of inflammatory cytokines in rats subjected to ischemia/reperfusion, reduced serum expression of TNF-α, IL-6, IL-8, etc., thus, alleviating inflammatory factor-induced cardiomyocyte injury and improving immune function (Wang et al., 2015). Research by Xuan Liu et al. discovered that SMI inhibited MCP-1, which indicated that SMI might be important in the inhibition of monocyte and macrophage activation (Liu et al., 2015). Lihua Du discovered that SMI significantly increased the weights of thymus and spleen in mice, raised serum IgG levels and the number of T cells, enhanced the phagocytic function of macrophages and had a significant immunomodulatory effect.

### Clinical Research on SMI

SFI has often been used to treat shock caused by various factors, COPD, systematic inflammatory response syndrome and other diseases in clinical practice (see **Table 3**). SMI has been recommended in China’s SARS Diagnosis and Treatment Scheme (Version 2004) and MERS Diagnosis and Treatment Scheme (Version 2015).

Biao Deng et al. studied 71 patients with shock and found that SMI in combination with conventional Western medicine had definite therapeutic efficacy, shortened the course of disease, reduced the length of hospital stay, and lowered the fatality rate (Deng et al., 2006). Hefeng Qin observed 68 patients with infectious shock and found that SMI had good clinical efficacy. It significantly improved CRP, PCT and TNF-α serum levels, and shortened the recovery time of vital signs with few adverse reactions (Qin, 2014). Wang Xian’an et al. observed 80 patients treated for infectious shock, and discovered that SMI in combination with ulinastatin had a significant therapeutic effect, enhancing immune function, and alleviating the inflammatory response (Wang et al., 2017). X. Huang et al. evaluated 23 RCTs with a total of 1,804 participants to study the impact of SMI on COPD. The results showed that SMI not only increased the total clinical response rate, but also improved pulmonary function, blood gas, and IgG indexes, and shortened the time for disappearance of lung rales. The results indicated that SMI in combination with Western medicine might have a positive effect in the treatment of COPD (Huang et al., 2019). Zongjun Fang et al. studied 38 patients with COPD. The control group (18 cases) received conventional Western medicine, while 20 cases (the treatment group) received SMI in addition. The results showed that patients in the treatment group had better vital capacity, forced expiratory volume in 1 s (FEV1), maximal breathing capacity (MBC), maximal inspiratory pressure (MIP), load breathing time, arterial blood gas analysis, and Burp dyspnea scores than the control group or the pre-treatment patients. The treatment group also had significantly improved respiratory function and clinical symptoms (Fang et al., 1998). Changxing Guo et al. randomized 33 patients with systemic inflammatory response syndrome into a conventional Western medicine treatment group (15 cases) and SMI + conventional treatment group (18 cases). After treatment, patients in the SMI treatment group had increased prostacyclin PGI2 and PGI2/thromboxane A2 (TXA2) in blood to a certain extent compared to patients in the conventional treatment group. Patients in the SMI group also had decreased levels of TXA2, atrial natriuretic peptide (ANP) and endothelin, and there were significant differences between the two groups. The results indicated that SMI could play an active role in improving microcirculation, protecting organ functions, and preventing further occurrence and development of systemic inflammatory response syndrome (Guo et al., 2004).

### Usage and Dosage of SMI

Intravenous drip, 20–60 ml diluted with 250–500 ml of 5% glucose injection.

### Adverse Reactions of SMI

The adverse reactions mainly include immediate hypersensitivity, predominantly skin allergy manifested by itching, rash, systematic urticaria, and then allergic shock; there may also be serious abdominal distension, corneal edema, abnormal vision, hypotension, ascending vascular pain, acute hepatic damage, sinus arrest, and drug fever.

### SMI Precautions

1) Not to be used in newborns, infants, pregnant women, or those with an allergic constitution. 2) Not for administration by intravenous injection. The administration speed should not be too fast. In those receiving the drug for the first time, the initial administration should be at 15 drips/min for 10 min. If there are no abnormalities, the speed of administration can be increased to normal, which is generally controlled at 40–50 drips/min. 3) This drug has a pressor response, and blood pressure should be monitored in hypertensive patients.

## Relevant Information on the Clinical Application of AGNHP

### Recommended Therapeutic Regimens

AGNHP has been recommended in 21 therapeutic regimens of COVID-19 in China (see detailed information in **Tables 1** and **2**).

### Ingredients of AGNHP

*Curcuma kwangsiensis* S.G.Lee & C.F.Liang (Yujin), Calculus Bovis (Niuhuang), Cornu Bubali (Shuiniujiao), *Coptis chinensis* Franch. (Huanglian), Cinnabaris (Zhusha), Moschus (Shexiang), Margarita (Zhenzhu), Realgar (Xionghuang), *Scutellaria baicalensis* Georgi (Huangqin), *Gardenia jasminoides* J.Ellis (Zhizi), and *Cinnamomum camphora* (L.) J.Presl (Bingpian). Basic information on AGNHP is provided in the **Supplementary Table**.

### Indications for the Treatment of COVID-19 With AGNHP

AGNHP is used for the syndrome of epidemic toxin lung closure and inner blocking causing collapse in the progressive stage of COVID-19 (critical case). Indicative symptoms are hyper-pyretic convulsions, coma and delirium, difficulty breathing, and dysphoria.

### Progress of Pharmacological Research on AGNHP

Modern pharmacological studies have shown that AGNHP has antipyretic, analgesic, anti-inflammatory, and neuroprotective effects (see **Table 3**).

Zuguang Ye et al. discovered that AGNHP could significantly reduce the body temperature of hyperpyrexic rabbits in a fever model induced by intravenous injection of typhoid Vi polysaccharide vaccine in rabbit ear (Ye et al., 2003). Feng Zhang, Kunjie Zhu et al. found in an LPS-induced intracerebral inflammation model that AGNHP antagonized the toxic effect of LPS on dopaminergic neurons, inhibited release of superoxide radical, and reverse changes in cortical monoamine neurotransmitters. It was speculated that its impact on cortical monoamine neurotransmitters might be one of the mechanisms by which AGNHP promoted consciousness in LPS brain damage (Zhang F. et al., 2010; Zhu and Sun, 2014). Research by Dan Zhang et al. showed that AGNHP lowered serum LPS and lung myeloperoxidase (MPO) levels in a rat model of sepsis (Zhang et al., 2009). Yishan Tang et al. found that AGNHP lowered total LDH activity in serum and brain tissue, and changed the percentage of isomerase in a rat pertussis-induced infectious cerebral edema model (Tang et al., 2005). Fan Q et al. discovered that AGNHP had anti-atherosclerotic effects in the high fat diet-induced ApoE^−/−^ mouse model at early- and mid-stage *via* regulation of Th17/Treg balance. It inhibited chronic inflammation, reduced plaque collagen fibers, and reduced inflammatory cell infiltration (Fan et al., 2020).

### Clinical Research on AGNHP

Modern clinical studies have shown that AGNHP has therapeutic effects against hyperpyrexia, coma caused by severe infectious diseases, and viral encephalitis and severe pneumonia of infants (see **Table 3**).

Yueming Feng et al. conducted a systematic evaluation and found that AGNHP could be used to promote consciousness of coma patients with acute cerebral infarction (ACI) and improve neurologic function. This may be due to the ability of AGNHP to alleviate the inflammatory response, reduce cerebral edema, and promote recovery of neurologic function (Feng and Yang, 2015). Haijun Zhang et al. discovered through clinical observation that AGNHP could be used to treat viral encephalitis in children. AGNHP rapidly reduced body temperature, prevented convulsions, promoted consciousness, and alleviated cerebral edema and brain cell damage (Zhang and Dong, 2014). Research by Zhulin Zhuo et al. found that AGNHP with the adjuvant, Ribavirin was efficacious in acute severe viral pneumonia of children, significantly reduced PCT and improved immune function (Zhuo and Wen, 2017). Yanling Shi discovered through clinical observation that AGNHP in combination with sodium phosphate improved anoxic conditions and myocardial damage in patients with neonatal asphyxia and myocardial damage (Shi, 2019). Xie Long et al. in a study of 70 patients with ACI and central hyperpyrexia found that combined use of conventional Western medicine and AGNHP reduced the duration of fever and significantly improved the prognosis (Long and Wu, 2014). Hanwei Liu et al. conducted a systematic review of relevant literature on AGNHP treatment of ACI and cerebral hemorrhage. The results showed that adjuvant treatment with ANP (AGNHP) appeared to improve the total response rate and neurologic deficit score in patients with ACI and acute intracerebral hemorrhage (AIH) (Han et al., 2019). Research by Ma et al. showed that ANP had a moderating effect on Th1/Th2 in cerebral infarction patients (Ma and Zhou, 2015).

### Usage and Dosage of AGNHP

Oral administration. 3 g, once a day.

### Adverse Reactions of AGNHP

Overdose administration might cause mercurial nephrosis or allergic reaction and other adverse reactions. Improper use of this product might cause hypothermia.

### AGNHP Precautions

1) Nasogastric administration can be used in patients unable to take orally because of high fever and coma. The pills can be dissolved in warm but not hot water. The water or decoction used to dissolve the pills should be controlled at 40–60°C. The use of boiling water is forbidden for two reasons: first, to avoid increased decomposition of realgar and cinnabar by high temperature, and reduce generation of the highly toxic arsenic trioxide, free arsenium, and mercury. Research has shown that arsenic trioxide in realgar preparations is not significantly changed below 60°C but begins to increase at 80°C. The decoction used for dissolving AGNHP should therefore not exceed 60°C secondly, musk, borneol and other aromatic substances are volatile. Boiling water could result in excess volatilization, thus, reducing efficacy. 2) Must not be used with nitrate, nitrite, ferrite or sulfate drugs. 3) Not for use in pregnant women. 4) It contains cinnabar and realgar, and should not be taken at high doses for long periods. Should be used with caution in those with hepatic and renal dysfunction. (5) It contains musk, so athletes should use with caution.

## Discussion and Conclusions

It is a critical moment in the battle to defeat the current outbreak of novel coronavirus. For this specific indication, rapid performance of TCM can contribute as an alternative measure. TCM can effectively prevent the disease from transforming into severe and critical disease. In severe cases, TCM has won time for recovery by improving symptoms (The State Council Information Office of the People’s Republic of China, 2020). Treatment practice for COVID-19 has shown that early intervention with TCM is an important method to improve cure rate, shorten the course of disease, delay disease progression and reduce mortality rate. For example, the total response rate of Qingfei Paidu Decoction was more than 90% in Shanxi and Hebei provinces (He et al., 2020).

CPMs have played an important role in preventing and treating epidemic diseases in China because they are convenient to use, easily stored and cost-effective. The positive role of CPMs has been emphasized in the “Diagnosis and Treatment of COVID-19 (Trial Version 7)” and other therapeutic regimens. During the medical observation period and early stage of COVID-19, HXZQC, LHQWC, SFJDC, and JHQGG can be selected according to different clinical manifestations. At the same time, they can also promote immunity against the virus. For severe and critical disease, the choice shall be made according to different syndromes during clinical treatment. For viral infections combined with mild bacterial infections, XYPI and TRQI can be used; for high fever with disturbance of consciousness, AGNHP can be used; for systemic inflammatory response syndrome or multiple organ function failure, XBJI is recommended; SMI can be used for immunosuppression; and SFI can be used for shock. Furthermore, the reason that TCM works is not only because it inhibits the virus, but also because it might block infection, regulate the immune response, inhibit the inflammatory storm, and promote repair of the body. Moreover, the prevention and control measures of COVID-19 have fully reflected the ideology of “preventive treatment of disease”.

Physicians should pay attention to the reasonable application of CPMs to treat COVID-19. Severe patients are prone to septic shock, and liver and kidney dysfunction. In patients with related underlying diseases, drug metabolism and clearance are reduced. Treatment options: hepato-renal toxic drugs should be avoided to reduce the risk of drug accumulation and poisoning. For example, AGNHP contains cinnabar and realgar, and should not be taken for a long time. People with liver and kidney dysfunction should use with caution. TRQI should be carefully selected because it aggravates liver and kidney function; LHQWC and JHQGG contain ephedrae herba (Mahuang), and doctors need to monitor patients’ blood pressure, heart condition and combined use of antihypertensive drugs. In clinical application of XBJI, SFI and other traditional Chinese medicine injections, attention should be paid to the choice of solvent and the interval between infusions with other drugs. As is well known, clinicians use CPMs under the guidance of the theory of TCM. Foreign doctors and patients wishing to use CPMs to treat COVID-19 should exercise caution, especially in countries where they may be used incorrectly without the knowledge of TCM theory.

There are some limitations within this paper. First, as there is little direct clinical evidence for the prevention of COVID-19, the reported studies are from previous reports on the prevention of SARS, MERS, H7N9, and H1N1 influenza by CPMs, which can only be considered as indirect evidence in respect of the current outbreak. Secondly, the programs for prevention of COVID-19 were issued shortly after the outbreak. Chinese medicine experts suggested CPMs to treat COVID-19 based on their previous experience in the prevention and treatment of similar diseases combined with their initial understanding of the disease. The actual effects of these programs need to be verified in clinical application, and updated and improved according to the evidence of new research on COVID-19.

For future studies, we recommend prospective cohort studies, RCTs or registry studies to evaluate the effect of CPMs in prevention of COVID-19. Some clinical trial protocols to treat COVID-19 using the top 10 CPMs are ongoing (see **Table 4**). At present, since COVID-19 has not yet been controlled, a series of prospective population studies with rigorous design and large sample should commence with protocol registration, and implementation in a timely manner, to produce reliable evidence for CM prevention of COVID-19 or similar emerging respiratory infectious diseases in the future.

**Table 4:** Registration information on clinical trial protocols for the top 10 CPMS in the treatment of COVID-19.

CPMS	Registration Number	Registration Date	Clinical research unit	Registration title
XYPJ	ChiCTR2000029756	2020/2/12	Renmin Hospital of Wuhan University (Wuhan, China)	Clinical study of nebulized Xiyanping injection in the treatment of novel coronavirus pneumonia (COVID-19)
	ChiCTR2000030117	2020/2/23	Jiangxi Qingfeng Pharmaceutical Co., Ltd. (Ganzhou, China)	A multicenter, randomized, open, parallel controlled trial for the evaluation of the effectiveness and safety of Xiyanping injection in the treatment of common type novel coronavirus pneumonia (COVID-19)
	ChiCTR2000030218	2020/2/25	Fifth People’s Hospital of Ganzhou (Ganzhou, China)	Study of Pinavir/Ritonavir tablets (Trade Name: Kelizhi) Combined with Xiyanping injection for novel coronavirus pneumonia (COVID-19)
LHQWC	ChiCTR2000029433	2020/2/1	Hebei Yiling Hospital (Shijiazhuang, China), Renmin Hospital of Wuhan University (Wuhan, China)	A randomized, open-label, blank-controlled trial for Lian-Hua Qing-Wen Capsule/granule in the treatment of suspected novel coronavirus pneumonia (COVID-19)
	ChiCTR2000029434	2020/2/1	Hebei Yiling Hospital (Shijiazhuang, China), Renmin Hospital of Wuhan University (Wuhan, China)	A randomized, open-label, blank-controlled trial for Lian-Hua Qing-Wen Capsule/granule in the treatment of novel coronavirus pneumonia (COVID-19)
TRQI	ChiCTR2000029432	2020/2/1	The First Affiliated Hospital of Guangzhou University of Chinese Medicine (Guangzhou, China)	A real world study for the efficacy and safety of large dose Tanreqing injection in the treatment of patients with novel coronavirus pneumonia (COVID-19)
	ChiCTR2000029813	2020/2/14	Shanghai Public Health Clinical Center (Shanghai, China)	Clinical trial for Tanreqing capsules in the treatment of novel coronavirus pneumonia (COVID-19)
XBJI	ChiCTR2000029381	2020/1/27	The First Affiliated Hospital of Guangzhou Medical University (Guangzhou, China)	A prospective comparative study for Xue-Bi-Jing injection in the treatment of novel coronavirus pneumonia (COVID-19)
	ChiCTR2000030388	2020/3/1	Jingzhou First People’s Hospital (Jingzhou, China)	Efficacy and safety of Xue-Bi-Jing injection in the treatment of severe cases of novel coronavirus pneumonia (COVID-19)
SFJDC	ChiCTR2000030043	2020/2/21	Peking University Third Hospital (Beijing, China)	Shen-Fu injection in the treatment of severe novel coronavirus pneumonia (COVID-19): a multicenter, randomized, open-label, controlled trial

## Author Contributions

WZ and ZF wrote the manuscript. HW, YY, LW, NS, GS and YS searched for related articles. YC, XL, and GG proofread the manuscript. SX guided the writing and critically revised the manuscript. All authors have read and approved the manuscript.

## Funding

This work was supported by the National Administration of Traditional Chinese Medicine “twelfth five-year” key clinical pharmacy construction project (No.ZP0101YX001), the National of Traditional Chinese Medicine Innovative Talents Training Project of China and the Fundamental Research Funds for the Central Universities of China (No.20720200012).

## Conflict of Interest

The authors declare that the research was conducted in the absence of any commercial or financial relationships that could be construed as a potential conflict of interest.

## Abbreviations

COVID-19, Coronavirus Disease 2019; CPMs, Chinese Patent Medicines; TCM, Traditional Chinese Medicine; HXZQC, Huoxiang Zhengqi capsule; LHQWC, Lianhua Qingwen capsule; JHQGG, Jinhua Qinggan granule; SFJDC, Shufeng Jiedu capsule; TRQI, Tanreqing injection; XYPI, Xiyanping injection; XBJI, Xuebijing injection; SFI, Shenfu injection; SMI, Shengmai injection; AGNHP, Angong Niuhuang pill; WHO, World Health Organization; ICU, intensive care unit; MODS, multiple organ dysfunction syndromes; AIV, avian influenza virus; LPS, lipopolysaccharide; IFN-γ, interferon-γ; IgG, immunoglobulin G; IBS, irritable bowel syndrome; IL-1, interleukin-1; IL-2, interleukin-2; IL-6, interleukin-6; IL-8, interleukin-8; IL-10, interleukin-10; IL-17, interleukin-17; IL-1α, interleukin-1α; IL-1β, interleukin-1β; TNF-α, tumor necrosis factor alpha; MCP-1, macrophage chemotactic factor-1; 5-HT, 5-hydroxytryptamine; LDH, lactate dehydrogenase; CRP, C-reactive protein; PCT, procalcitonin; AST, aspartate aminotransferase; ALT, glutamate transaminase; MDA, malondialdehyde; ATP, adenosine triphosphate; SOD, superoxide dismutase; ROS, reactive oxygen species; CPE, cytopathic effect; WBC, white blood cell; Th, T helper cell; NK, natural killer; MAPK, mitogen-activated protein kinase; PI3K, phosphatidylinositol 3 kinase; NF-κB, nuclear factor kappa B; HLA-DR, human leukocyte antigen-DR; ICAM-1, intracellular adhesion molecule 1; VCAM-1, vascular cell adhesion molecule 1; TXA2, thromboxane A2; ANP, atrial natriuretic peptide; BALF, bronchoalveolar lavage fluid; URI, upper respiratory infection; SARS, severe acute respiratory syndrome; MERS, Middle East respiratory syndrome; RCTs, randomized controlled trials; CAP, community acquired pneumonia; COPD, chronic obstructive pulmonary disease; AECOPD, acute exacerbation of chronic obstructive pulmonary disease; MRSA, methicillin-resistant *Staphylococcus aureus*; SIRS, systemic inflammatory response syndrome; APACHE-II, acute physiology and chronic health evaluation II.

## References

[B1] Ai Y. H. Peng L. Zhang L. N. (2006). Protective effect of Shenfu injection on endotoxin induced acute lung injury. Chin. J. Crit. Care Med. 26, 285–286. 10.7666/d.y736206

[B2] Bao Y. Y. Gao Y. J. Shi Y. J. Bao L. Yao R. M. Mao X. (2019). Study on Broad-spectrum antiviral effect of Shufeng Jiedu Capsules. J. New. Chin. Med. 51, 5–8. 10.13457/j.cnki.jncm.2019.12.002

[B3] Chen Y. Q. Gong B. L. Zhang Y. Xu Q. X. (2011). Effects of Xuebijing on patients with acute exacerbation chronic obstructive pulmonary disease. Chin. Gen. Prac. 14, 550–553. 10.3969/j.issn.1007-9572.2011.05.032

[B4] Chen X. Feng Y. X. Shen X. Y. Pan G. X. Fan G. W. Gao X. M. (2018). Anti-sepsis protection of Xuebijing injection is mediated by differential regulation of pro-and anti-inflammatory Th17 and T regulatory cells in a murine model of polymicrobial sepsis. J. Ethnopharmacol. 211, 358–365. 10.1016/j.jep.2017.10.001 28987599

[B5] Cheng D. Z. Wang W. J. Li Y. Wu X. D. Zhou B. Song Q. Y. (2020). Analysis of 51 cases of novel coronavirus pneumonia treated with traditional Chinese medicine Lianhua Qingwen: a multicenter retrospective study. Tianjin J. Tradit. Chin. Med. 37, 509–516. 10.11656/j.issn.1672-1519.2020.05.06

[B6] Chinese Medical Association and China Association of Chinese Medicine (2004). Guideline on diagnosis and treatment of SARS (2004 edition). Mod. Pract. Med. 16, 119–126. 10.3969/j.issn.1671-0800.2004.02.033

[B7] Cui W. W. Jin X. Zhang Y. F. Chang L. P. Wang H. T. (2016). Effects of Lianhua Qingwen Capsules on IKK/IκB/NF-κB signal pathway in the mouse with LPS-induced acute lung injury. Chin. Tradit. Pat. Med. 37, 954–958. 10.3969/j.issn.1001-1528.2015.05.006

[B8] Deng B. Huang X. K. Li Q. (2006). Clinical observation of Shengmai Injection in treatment of shock. Chin. J. Postgraduat. Med. 29, 26–27. 10.3760/cma.j.issn.1673-4904.2006.03.010

[B9] Ding Y. W. Zeng L. J. Li R. F. Chen Q. Y. Zhou B. X. Chen Q. L. (2017). The Chinese prescription lianhuaqingwen capsule exerts anti-influenza activity through the inhibition of viral propagation and impacts immune function. BMC Complement. Altern. Med. 17, 130. 10.1186/s12906-017-1585-7 28235408 PMC5324200

[B10] Dong L. Xia J. W. Gong Y. Chen Z. Yang H. H. Zhang J. (2014). Effect of lianhuaqingwen capsules on airway inflammation in patients with acute exacerbation of chronic obstructive pulmonary disease. Evid. Based. Complement. Alternat. Med. 2014, 637969. 10.1155/2014/637969 24971150 PMC4058171

[B11] Du L. H. Deng X. R. Zhang C. J. Xie P. L. Yang L. (2001). Effect of Shengmai Injection on the immune function of mice. Shanghai J. Immune 21, 247–248. 10.3969/j.issn.1001-2478.2001.04.020

[B12] Duan Z. P. Jia Z. H. Zhang J. Liu S. Chen Y. Liang L. C. (2011). Natural herbal medicine Lianhuaqingwen capsule anti-influenza A (H1N1) trial: a randomized, double blind, positive controlled clinical trial. Chin. Med. J. 124, 2925–2933. 10.3760/cma.j.issn.0366-6999.2011.18.024 22040504

[B13] Duan C. Xia W. G. Zheng C. J. Sun G. B. Li Z. L. Li Q. L. (2020). Clinical observation of Jinhua Qinggan Granule to treat COVID-19. J. Tradit. Chin. Med. 61, 1–5.

[B14] Fan Q. Liu Y. Rao J. Zhang Z. Xiao W. Zhu T. (2020). Anti-atherosclerosis effect of Angong Niuhuang Pill via regulating Th17/Treg immune balance and inhibiting chronic inflammatory on ApoE^-/-^ mice model of early and mid-term. Front. Pharmacol. 10, 1584. 10.3389/fphar.2019.01584 32082145 PMC7005527

[B15] Fang Z. J. Jiang H. M. Wang L. H. (1998). Therapeutic effect of Shengmai Injection on respiratory function in chronic obstructive pulmonary disease. Chin. J. Integr. Tradit. West. Med. 9, 520. 10.3321/j.issn:1003-5370.1998.09.040 11475725

[B16] Feng Y. M. Yang H. (2015). Study on revival effect of Angong Niuhuang Pills in treating coma caused by acute cerebral infarction. Chin. J. Exper. Tradit. Med. Form. 21, 179–182. 10.13422/j.cnki.syfjx.2015060179

[B17] Fisher D. Heymann D. (2020). Q&A: The novel coronavirus outbreak causing COVID-19. BMC Med. 18, 57. 10.1186/s12916-020-01533-w 32106852 PMC7047369

[B18] Gao L. N. Lv J. Wang Z. F. Yu D. D. Sun M. H. (2019). Meta-analysis of randomized controlled trials on effect of Tanreqing Injection combined with Western medicine on acute exacerbation of chronic bronchitis. Chin. J. Chin. Mater. Med. 44, 5313–5321. 10.19540/j.cnki.cjcmm.20190924.501 32237374

[B19] Gao Y. B. (2020). Giving full play to the advantages of integrated Chinese and Western medicine to win the battle against the epidemic. Sci. Tech. Indust. Chin. 370, 17–20. 10.16277/j.cnki.cn11-2502/n.2020.04.009

[B20] Guo C. X. Yang X. Y. Lin Z. F. Zhao L. Shan H. W. Chen D. C. (2004). Clinical observation on effects of Shengmai injection on plasma vasoactive mediators in patients with systemic inflammatory response syndrome. Chin. J. Integr. Tradit. West. Med. Intensive Crit. Care 11, 239–241. 10.3321/j.issn:1008-9691.2004.04.008

[B21] Han W. Liu Y. Y. Peng F. P. Mao J. J. Hu X. J. Li D. (2019). Angong Niuhuang Pill as adjuvant therapy for treating acute cerebral infarction and intracerebral hemorrhage: A meta-analysis of randomized controlled trials. J. Ethnopharmacol. 237, 307–313. 10.1016/j.jep.2019.03.043 30910581

[B22] Han X. P. (2016). Effect of oseltamivir phosphate assisted Huoxiang Zhengqi Liquid on the treatment of influenza. Chin. J. Mod. Drug Appl. 10, 139–141. 10.14164/j.cnki.cn11-5581/r.2016.18.092

[B23] He Y. H. Zhao H. Y. Liu Z. L. Lu C. Luo X. J. Lin S. Q. (2006). Effects of huoxiangzhengqi liquid on enteric mucosal immune responses in mice with Bacillus dysenteriae and Salmonella typhimurium induced diarrhea. World J. Gastroenterol. 12, 7346–7349. 10.3748/wjg.v12.i45.7346 17143954 PMC4087496

[B24] He Q. H. Liu Y. K. Sun X. R. Sun Y. K. Sun G. R. (2020). The significance and role of “Qingfei Paidu Decoction” recommended by the National Health Commission and the National Administration of Traditional Chinese Medicine. J. Tradit. Chin. Med. 61, 892–832. 10.13288/j.11-2166/r.2020.10.001

[B25] Hu K. Guan W. J. Bi Y. Zhang W. Li L. J. Zhang B. L. (2020). Efficacy and safety of Lianhuaqingwen capsules, a repurposed Chinese herb, in patients with coronavirus disease 2019: A multicenter, prospective, randomized controlled trial. Phytomedicine 76, 153242. 10.1016/j.phymed.2020.153242 PMC7229744 33867046

[B26] Huang H. F. Chen Y. Zhu X. X. Chen T. (2016). Traditional Chinese medicine intervention regulates brain-gut peptides of irritable bowel syndrome. Chin. J. Exper. Tradit. Med. Form. 22, 208–217. 10.13422/j.cnki.syfjx.2016110208

[B27] Huang X. Y. Duan X. J. Wang K. H. Wu J. R. Zhang X. M. (2019). Shengmai Injection as an adjunctive therapy for the treatment of chronic obstructive pulmonary disease: a systematic review and meta-analysis. Complement. Ther. Med. 43, 140–147. 10.1016/j.ctim.2019.01.020 30935521

[B28] Huang C. L. Wang Y. M. Li X. W. Ren L. L. Zhao J. P. Hu Y. (2020). Clinical features of patients infected with 2019 novel coronavirus in Wuhan, China. Lancet 395, 497–506. 10.1016/S0140-6736(20)30183-5 31986264 PMC7159299

[B29] Jiang H. L. Mao B. Zhong Y. Q. Yang H. M. Fu J. J. (2009). Tanreqing Injection for community-acquired pneumonia: a systematic review of randomized evidence. J. Chin. Integr. Med. 7, 9–19. 10.3736/jcim20090102 19134452

[B30] Jin H. Chen Yi. Ding C. J. Lin Y. P. Chen Y. L. Jiang D. X. (2018). Microcirculatory disorders and protective role of Xuebijing in severe heat stroke. Sci. Rep. 8, 4553. 10.1038/s41598-018-22812-w 29540802 PMC5852149

[B31] Li P. T. Zhang N. Zhu X. L. Xu Y. Xie L. J. Wang B. (2005). Protective effects of Tanreqing on lipopolysaccharide-induced acute lung injury in rats. Chin. Pharm. J. 40, 518–521. 10.3321/j.issn:1001-2494.2005.07.013

[B32] Li W. Mao B. Wang G. Wang L. Chang J. Zhang Y. (2010). Effect of Tanreqing Injection on treatment of acute exacerbation of chronic obstructive pulmonary disease with Chinese medicine syndrome of retention of phlegm and heat in Fei. Chin. J. Integr. Med. 16, 131–137. 10.1007/s11655-010-0131-y 20473738

[B33] Li G. Q. Zhao J. Tu Z. T. Hu J. Q. (2013). Treating influenza patients of wind-heat affecting fei syndrome by Jinhua Qinggan Granule: a double-blinded randomized control trial. Chin. J. Integr. Tradit. West. Med. 33, 1631–1635. 10.7661/CJIM.2013.12.1631 24517059

[B34] Li C. Y. Zhou X. Y. Wu S. Q. Gong M. J. Wang S. M. Liang S. W. (2017). Urinary metabonomics study on effect of Huoxiang Zhengqi Oral Liquid in rats with dampness obstructing spleen-stomach syndrome. Tradit. Chin. Drug Res. Clin. Pharm. 28, 95–99. 10.19378/j.issn.1003-9783.2017.04.018

[B35] Li Y. M. Chang N. W. Han Y. Q. Zhou M. G. Gao J. Hou Y. Y. (2017). Anti-inflammatory effects of Shufengjiedu capsule for upper respiratory infection via the ERK pathway. Biomed. Pharmacother. 94, 758–766. 10.1016/j.biopha.2017.07.118 28802227

[B36] Li C. Y. Wang P. Zhang L. Li M. Lei X. Liu S. (2018). Efficacy and safety of Xuebijing injection (a Chinese patent) for sepsis: A meta-analysis of randomized controlled trials. J. Ethnopharmacol. 224, 512–521. 10.1016/j.jep.2018.05.043 29860133

[B37] Li Q. Yin J. Ran Q. S. Yang Q. Liu L. Zhao Z. (2019). Efficacy and mechanism of Lianhua Qingwen Capsules (LHQW) on chemotaxis of macrophages in acute lung injury (ALI) animal model. Chin. J. Chin. Mater. Med. 44, 2317–2323. 10.19540/j.cnki.cjcmm.20190210.001 31359658

[B38] Li R. F. Hou Y. L. Huang J. C. Pan W. Q. Ma Q. H. Shi Y. X. (2020). Lianhuaqingwen exerts anti-viral and anti-inflammatory activity against novel coronavirus (SARS-CoV-2). Pharmacol. Res. 156, 104761. 10.1016/j.phrs.2020.104761 32205232 PMC7102548

[B39] Li T. T. Qian Y. M. Miao Z. L. Zheng P. Y. Shi T. Jiang X. R. (2020). Xuebijing Injection alleviates Pam3CSK4-induced inflammatory response and protects mice from sepsis caused by methicillin-resistant staphylococcus aureus. Front. Pharmacol. 11, 104. 10.3389/fphar.2020.00104 32153410 PMC7047170

[B40] Li J. (2013). Clinical research of Shenfu Injection on duration of mechanical ventilation in patients with respiratory failure. J. Liaoning Univ. Tradit. Chin. Med. 15, 160–162. 10.13194/j.jlunivtcm.2013.02.162.lij.077

[B41] Li G. M. (2015). The effect of viral pneumonia linc treatment observation Xiyanping. Chin. Cont. Med. Educat. 7, 256–257. 10.3969/j.issn.1674-9308.2015.06.213

[B42] Li T. H. (2019). Clinical evaluation of Lianhua Qingwen Capsule in the treatment of acute upper respiratory infection. Guide Chin. Med. 17, 199–200. 10.15912/j.cnki.gocm.2019.12.152

[B43] Lian X. Ling C. Wang C. Q. Yue J. B. Li Y. Q. Zhou W. J. (2018). Clinical efficacy and safety of Tanreqing Injection for pulmonary infection in patients with tuberculosis: A meta-analysis. J. Altern. Complement. Med. 24, 1051–1062. 10.1089/acm.2018.0020 30124323

[B44] Liang J. Z. Luo Q. H. Fang Y. S. Gu Z. J. Huang Y. Q. (2013). Clinical efficacy on Tanreqing Injection for curing hand-foot-mouth disease: A meta-analysis. Chin. J. Evid. Based. Med. 13, 1446–1454. 10.7507/1672-2531.20130248

[B45] Lin L. Zhan L. Y. (2010). The protective effect of shen-Fu on lipopolysaccharide-induced acute lung injury in rats. J. Pract. Med. 26, 942–944. 10.3969/j.issn.1006-5725.2010.06.022

[B46] Lin L. N. Zhang S. G. Wang W. T. Qiu X. X. Dai Y. Y. Wang Q. (2007). Effect of Shengmai injection on pneumocyte apoptosis during pulmonary ischemia /reperfusion injury in rabbits. Chin. J. Integr. Tradit. West. Med. Intensive Crit. Care 14, 361–363, 395. 10.3321/j.issn:1008-9691.2007.06.011

[B47] Liu X. W. Li Y. Y. (2015). The study on therapeutic effect of Xiyanping injection for acute upper respiratory tract infection. Chin. Prac. Med. 10, 125–126. 10.14163/j.cnki.11-5547/r.2015.02.087

[B48] Liu H. F. Zhao Y. Y. Wei F. (2009). Effect of pulse-activating injection on the expression of nuclear factor-kappa B and iNOS in lung tissue of acute paraquat poisoned rats. Lishizhen Med. Mater. Med. Res. 20, 1930–1932. 10.3969/j.issn.1008-0805.2009.08.045

[B49] Liu Y. Shi H. Jin Y. H. Gao Y. J. Shi Y. J. Liu F. F. (2010). Experimental pharmacodynamic research in vivo of Shufengjiedu Capsule on treatment and prevention of influenza. World J. Integr. Tradit. West. Med. 5, 107–110. 10.13935/j.cnki.sjzx.2010.02.020

[B50] Liu M. W. Su M. X. Zhang W. Wang Y. Q. Chen M. Wang L. (2014). Protective effect of Xuebijing injection on paraquat-induced pulmonary injury via down-regulating the expression of p38 MAPK in rats. BMC Complement. Altern. Med. 14, 498. 10.1186/1472-6882-14-498 25511395 PMC4301062

[B51] Liu X. Li Z. Hua S. Y. Hou M. M. Wang Y. Kang L. Y. (2015). Polypharmacology of Shengmai Injection against rat myocardial ischemia and reperfusion injury. Chin. Tradit. Pat. Med. 37, 251–255. 10.3969/j.issn.1001-1528.2015.02.003

[B52] Liu W. Jiang H. L. Cai L. L. Yan M. Dong S. J. Mao B. (2016). Tanreqing Injection attenuates lipopolysaccharide-induced airway inflammation through MAPK/NF-κB signaling pathways in rats model. Evid. Based. Complement. Alternat. Med. 2016, 5292346. 10.1155/2016/5292346 27366191 PMC4913016

[B53] Liu W. Zhang X. Mao B. Jiang H. (2019). Systems pharmacology-based study of Tanreqing injection in airway mucus hypersecretion. J. Ethnopharmacol. 249, 112425. 10.1016/j.jep.2019.112425 31765763

[B54] Liu X. Ai F. Chu C. W. Chen X. Y. Guo J. F. Yang Y. (2019a). Improvement and anti-inflammation mechanism of Shenfu Injection on lung tissue in endotoxin shock model rats. Chin. Pharm. 30, 1492–1497. 10.6039/j.issn.1001-0408.2019.11.11

[B55] Liu X. Liu R. Z. Dai Z. F. Wu H. Lin M. Tian F. (2019b). Effect of Shenfu injection on lipopolysaccharide (LPS)-induced septic shock in rabbits. J. Ethnopharmacol. 234, 36–43. 10.1016/j.jep.2019.01.008 30641104

[B56] Liu L. L. Yuan L. F. Feng Y. Sun D. Liu W. S. Wang Y. J. (2020). Clinical study on combined scheme of Lianhuaqingwen Capsules and abidole in the treatment for coronavirus disease 2019. Guangdong Med. J. 41, 1–4. 10.13820/j.cnki.gdyx.20200913

[B57] Liu Z. H. (2009). The establishment of the animal model infected by Avian Influenza Virus in mice and the effect of interference Avian Influenza Virus of the Chinese Herbal Compound (Guangzhou: Guangzhou University of Chinese Medicine).

[B58] Liu Q. (2015). Study on the antiviral effect of mice infected by Xiyanping injection of human rhinovirus. Chin. J. Clin. Ration Drug Use 8, 23–24. 10.15887/j.cnki.13-1389/r.2015.23.012

[B59] Long X. Wu H. B. (2014). Clinical observation of Angong Niuhuang Pill in the treatment of acute cerebral infarction with hyperthermia. J. Emerg. Tradit. Chin. Med. 23, 1922–1923. 10.3969/j.issn.1004-745X.2014.10.069

[B60] Luo P. Zhou Z. X. (2017). Protective effects of xuebijing on the acute lung injury in rats. Chin. J. Appl. Physiol. 33, 132–135. 10.12047/j.cjap.5464.2017.034 29931920

[B61] Lv W. W. Zhu T. N. Qiu H. Hu T. Huang S. H. (2013). Pharmacodynamic study on antiviral and antibacterial effects of Shufeng Jiedu capsules in vitro. Tradit. Chin. Drug Res. Clin. Pharm. 24, 234–238. 10.3969/j.issn.1003-9783.2013.03.006

[B62] Lv S. J. Lai D. P. Wei X. Yan Q. Xia J. M. (2017). The protective effect of Shenfu injection against elderly severe pneumonia. Eur. J. Trauma. Emerg. S. 43, 711–715. 10.1007/s00068-016-0713-2 27458066

[B63] Lyu R. B. Wang W. J. Li X. (2020). Clinical Observation on s Lianhua Qingwen Granules Combined with Western Medicine Conventional Therapy in the Treatment of 63 Suspected Cases of Coronavirus Disease 2019. J. Tradit. Chin. Med. 61, . 655–. 659. 10.13288/j.11-2166/r.2020.08.003

[B64] Ma S. P. Wang X. Y. (2012). Huoxiang Zhengqi Powder combined with western medicine in the treatment of 80 children with rotavirus enteritis. Shaanxi J. Tradit. Chin. Med. 33, 1463. 10.3969/j.issn.1000-7369.2012.11.015

[B65] Ma J. J. Zhou C. X. (2015). Effect-of Angong Niuhuang Pill on Th1/Th2 of cerebral infarction patients of phlegm-heat obstructing orifices in China and Indonesia. Chin. J. Integr. Tradit. West. Med. 35, 287–289. 10.7661/CJIM.2015.03.0287 25951631

[B66] Ma Y. X. Guo Y. L. Liu J. Wang Y. G. (2015). Study on treatment of influenza a H1N1 induced severe pneumonia by Xuebijing Injection. World Chin. Med. 10, 243–246. 10.3969/j.issn.1673-7202.2015.02.023

[B67] Ma L. Huang Y. Hou Y. B. Xu J. Zhu Q. Liu J. (2018). Anti-inflammatory mechanism of Shufeng Jiedu Capsules in rat pneumonia model. Chin. Tradit. Herbal. Drugs 49, 4591–4595. 10.7501/j.issn.0253-2670.2018.19.019

[B68] Ma M. Cao B. Chang X. Y. Li W. H. Bao Y. Z. Zhao J. Q. (2019). The clinical study of the effects of Shenfu Injection on regulating traumatic acute lung injury in vascular endothelial cell function. Syst. Med. Pharmacol. 4, 36–38. 10.19368/j.cnki.2096-1782.2019.10.036

[B69] Ma L. Hou Y. B. Huang Y. Bi H. Y. Li X. Y. Ding L. F. (2019a). Regulation of components of Shufeng Jiedu Capsule on immune system of pneumonia model rats. Chin. Tradit. Herbal. Drugs 50, 3563–3568. 10.7501/j.issn.0253-2670.2019.15.007

[B70] Ma L. Huang Y. Hou Y. B. Zhang D. D. Zhang Y. M. Liu J. J. (2019b). Study on mechanism for immunoregulation of Shufeng Jiedu Capsule. Drug Evaluat. Res. 42, 1763–1768. 10.7501/j.issn.1674-6376.2019.09.009

[B71] National Health and Family Planning Commission of People’s Republic of China (2015). Guideline on diagnosis and treatment of Middle East respiratory syndrome (2015 version). Chin. J. Viral. Dis. 5, 352–354. 10.16505/j.2095-0136.2015.05.005

[B72] National Health and Family Planning Commission of People's Republic of China (2017). Guideline on diagnosis and treatment of human infection with avian influenza A (H7N9) virus (2017 version). Chin. J. Viral Dis. 7, 1–4. 10.16505/j.2095-0136.2017.01.001

[B73] National Health Commission of the People's Republic of China and National Administration of Traditional Chinese Medicine (2019). Protocol for diagnosis and treatment of influenza (2019 version). Chin. J. Clin. Infect. Dis. 12, 451–455. 10.3760/cma.j.issn.1674-2397.2019.06.003

[B74] National Health Commission of the People's Republic of China and National Administration of Traditional Chinese Medicine (2020). Guideline on diagnosis and treatment of COVID-19 (Trial 6th edition). Tianjin J. Tradit. Chin. Med. 37, 242–246. 10.11656/j.issn.1672-1519.2020.03.02

[B75] Nie Y. L. Fan B. Yan H. Tang C. S. Peng J. Guo N. (2012). Effects of cytokine content of Xiyanping Injection on acute lung injury induced by LPS in rat bronchoalveolar lavage. Chin. J. Basic Med. Tradit. Chin. Med. 18, 976–978.

[B76] Pan J. W. (2016). A systematic review about traditional Chinese medicine treatment of viral pneumonia (Guangzhou: Guangzhou University of Chinese Medicine).

[B77] Ping F. Li Z. S. Zhang F. R. Li D. X. Han S. Z. (2016). Effects of Lianhua Qingwen on pulmonary oxidative lesions induced by fine particulates (PM2.5) in rats. Chin. Med. Sci. J. 31, 233–238. 10.1016/s1001-9294(17)30006-8 28065220

[B78] Qi F. Liang Z. X. She D. Y. Yan G. T. Chen L. A. (2011). A clinical study on the effects and mechanism of Xuebijing Injection in severe pneumonia patients. J. Tradit. Chin. Med. 31, 46–49. 10.1016/s0254-6272(11)60011-3 21563507

[B79] Qi J. P. Qi X. Y. Wang X. J. (2016). Clinical efficacy of different doses of Jinhuaqinggan granule on influenza and serum levels of cytokines. Mod. Med. J. 44, 1664–1669. 10.3969/j.issn.1671-7562.2016.12.004

[B80] Qi R. H. Fang S. N. Li D. M. Zhang H. C. (2018). System evaluation and meta-analysis of Xiyanping injection in the treatment of adult viral pneumonia. Mod. Chin. Clin. Med. 25, 29–33. 10.3969/j.issn.2095-6606.2018.03.009

[B81] Qin H. F. (2014). Curative effect of Shengmai Injection in the treatment of septic shock. Chin. Med. Herald. 11, 86–89.

[B82] Qiu Z. L. Ye Y. P. Zhang N. (2012). Clinical efficacy of Shenfu injection in treating severe sepsis and its effects on serum levels of interleukin-6 and interleukin-10. Chin. J. Integr. Tradit. West Med. 32, 348–351. 10.7661/CJIM.2012.3.348 22686081

[B83] Qiu H. Li Z. X. Zhu T. N. Wu X. Lv W. W. Huang S. H. (2014). In-vivo experimental research of antiviral action of Shufeng Jiedu Capsule. Tradit. Chin. Drug Res. Clin. Pharm. 25, 14–17. 10.3969/j.issn.1003-9783.2014.01.05

[B84] Shi Y. L. (2019). Clinical effects of Angong Niuhuang Pills combined with sodium phosphocreatine on patients with asphyxia neonatorum and myocardial injury. Chin. Tradit. Pat. Med. 41, 2114–2117. 10.3969/j.issn.1001-1528.2019.09.019

[B85] Song Y. L. Yao C. Yao Y. M. Han H. Zhao X. D. Yu K. J. (2019). XueBiJing Injection versus placebo for critically III patients with severe community-acquired pneumonia: A randomized controlled trial. Crit. Care Med. 47, 734–743. 10.1097/CCM.0000000000003842 PMC6727951 31162191

[B86] Tang Y. S. Lin P. Y. Ou W. P. (2005). Effects of cinnabar and realgar in Angong Niuhuang powder on lactate dehydrogenase and its isoenzymes in rats with infectious cerebral edema. Chin. J. Integr. Tradit. West. Med. 25, 436–440. 10.3321/j.issn:1003-5370.2005.05.014 15957839

[B87] Tang S. W. Zhang Y. F. Liu K. J. Xu D. F. Wang H. R. Li Z. J. (2015). Effects of Lianhua Qingwen Capsule on lung tissue injury and expression of inflammatory cytokines in mice exposed to automobile exhaust. Chin. J. Exp. Tradit. Med. Form. 21, 139–143. 10.13422/j.cnki.syfjx.2015130139

[B88] Tao Z. G. Gao J. Y. Xue M. M. Yang W. Q. Zhang Y. P. Shen H. (2014). Shufeng Jiedu Capsule regulates LPS-induced acute lung injury via inhibiting MAPK/NF-κB pathways. Chin. J. Tradit. Chin. Med. Pharm. 29, 911–915.

[B89] Teng Y. Xiao J.,. R. Lin P. Zhang J. (2012). Application of Xuebijing in treatment of severe pneumonia and its effects on inflammatory factors and cellular immunity. Chin. J. Exp. Tradit. Med. Form. 18, 295–297. 10.13422/j.cnki.syfjx.2012.17.088

[B90] The State Council Information Office of the People’s Republic of China http://www.scio.gov.cn/xwfbh/xwbfbh/wqfbh/42311/42560/index.htm (accessed 20 Feb 2020).

[B91] Wang L. Qiu X. M. (2018). Evaluation on clinical efficacy of Shufengjiandu Capsules in fever patients accompanied with acute upper respiratory tract infection. Anti. Infect. Pharm. 15, 1406–1408. 10.13493/j.issn.1672-7878.2018.08-042

[B92] Wang L. Zhao F. Xu H. Liu K. (2008). The effects of Xiyanping injection on the release of pro-inflammatory cytokines in RAW 264.7 induced by LPS. Pharmacol. Clin. Chin. Mater. Med. 24, 36–39. 10.3969/j.issn.1001-859X.2008.01.018

[B93] Wang C. Cao B. Liu Q. Q. Zou Z. Q. Liang Z. A. Gu L. (2011). Oseltamivir compared with the Chinese traditional therapy maxingshigan-yinqiaosan in the treatment of H1N1 influenza: a randomized trial. Ann. Intern. Med. 155, 217–225. 10.7326/0003-4819-155-4-201108160-00005 21844547

[B94] Wang Y. Wang T. Hu J. J. Ren C. Y. Lei H. T. Hou Y. M. (2011). Anti-biofilm activity of TanReQing, a traditional Chinese medicine used for the treatment of acute pneumonia. J. Ethnopharmacol. 134, 165–170. 10.1016/j.jep.2010.11.066 21182923

[B95] Wang Z. Z. Yang Z. H. Grinchuk V. Zhu K. J. Shea-Donohue T. Zhao A. P. (2012). Mo2038 anti-inflammatory and intestinal function-modulating activities of a traditional chinese herbal formula Huoxiang Zhengqi. Gastroenterology 142, S–726. 10.1016/S0016-5085(12)62817-0

[B96] Wang C. L. Wu X. J. Xue M. M. Song Z. J. Yao C. L. Zhang Y. P. (2014). Combination of “Shufeng Jiedu Capsule” and antibiotics for the treatment of diabetes combined with pulmonary infection. Shanghai J. Tradit. Chin. Med. 48, 39–41, 48. 10.16305/j.1007-1334.2014.11.031

[B97] Wang C. Liu L. M. Song Z. Q. Dong Y. Z. Du Z. Y. Ning Z. C. (2015). Survey of active components in commonly-used Chinese materia medica injections and related Chinese materia medica for cardiovascular disease. Chin. Tradit. Herbal. Drugs 46, 2315–2328. 10.7501/j.issn.0253-2670.2015.15.024

[B98] Wang P. Liao X. Xie Y. M. Chai Y. Li L. H. (2016). Tanreqing injection for acute bronchitis disease: A systematic review and meta-analysis of randomized controlled trials. Complement. Ther. Med. 25, 143–158. 10.1016/j.ctim.2016.02.008 27062962

[B99] Wang X. A. Chen L. Wang Y. H. (2017). Clinical study on Shengmai Injection combined with ulinastatin in treatment of septic shock. Drugs Clin. 32, 249–252. 10.7501/j.issn.1674-5515.2017.02.021

[B100] Wang S. H. Liu J. F. Zhang Y. L. Dong Z. (2019). Systematic review of efficacy and safety of Lianhua Qingwen Capsules in treatment of viral influenza. Chin. J. Chin. Mater. Med. 44, 1503–1508. 10.19540/j.cnki.cjcmm.20190102.001 31090311

[B101] Wang T. L. (2015). Clinical effect of shufeng detoxification capsules on mild and moderate acute exacerbation patients with chronic obstructive pulmonary disease. Pract. J. Card. Cereb. Pneum. Vasc. Dis. 23, 149–151. 10.3969/j.issn.1008-5971.2015.07.048

[B102] Wang Z. H. (2016). Efficacy analysis of Shufeng Jiedu capsule in treating community-acquired pneumonia. World Chin. Med. 11, 1510–1512, 1516. 10.3969/j.issn.1673-7202.2016.08.032

[B103] Wen L. Zhou Z. G. Jiang D. X. Huang K. (2020). Effect of Xuebijing Injection on Inflammatory Markers and Disease Outcome of Coronavirus Disease 2019. Chin. Crit. Care Med. 32, 426–429. 10.3760/cma.j.cn121430-20200406-00386 32527346

[B104] Wu Z. McGoogan J. M. (2020). Characteristics of and important lessons from the coronavirus disease 2019 (COVID-19) outbreak in China: summary of a report of 72314 cases from the Chinese center for disease control and prevention. JAMA. 323, 1239–1242. 10.1001/jama.2020.2648 32091533

[B105] Wu X. Z. (2010). Clinical research on curative effect and safety about the curatio of agastachis powder for restoring healthy energy on diseases caused by exogenous pathogenic factor with moist type cold (Guangzhou: Guangzhou University of Chinese Medicine).

[B106] Xu S. H. Liu S. Y. (2010). Progress on pharmacological effect of Shengmai injection. Chin. Pharmaceut. Affairs 24, 405–407. 10.16153/j.1002-7777.2010.04.014

[B107] Yang Z. X. Fan T. B. Li J. (2014). Xiyanping Injection for severe pneumonia with syndrome of phlegm-heat obstructing lung. Beijing J. Tradit. Chin. Med. 33, 894–896. 10.16025/j.1674-1307.2014.12.004

[B108] Yang W. Liu J. Blažeković B. Sun Y. Ma S. Ren C. (2018). In vitro antibacterial effects of Tanreqing injection combined with vancomycin or linezolid against methicillin-resistant Staphylococcus aureus. BMC Complement. Altern. Med. 18, 169. 10.1186/s12906-018-2231-8 29848316 PMC5977505

[B109] Yao K. T. Liu M. Y. Li X. Huang J. H. Cai H. B. (2020). Retrospective clinical analysis on treatment of novel coronavirus-infected pneumonia with traditional Chinese medicine Lianhua Qingwen. Chin. J. Exp. Tradit. Med. Form. 26, 8–12. 10.13422/j.cnki.syfjx.20201099

[B110] Ye Z. G. Wang J. H. Liang A. H. Xue B. Y. Wang Y. S. Wang Z. M. (2003). Comparative studies on pharmacological effects of Angong Niuhuang pill with its simplified prescription. Chin. J. Chin. Mater. Med. 28, 636–639. 10.3321/j.issn:1001-5302.2003.07.014 15139109

[B111] Yin X. P. Xie Y. M. Zhi Y. J. Yang W. Wang Z. F. Huo J. (2015). Correlation analysis on combined medication with of Xiyanping injection in treatment of lung infection in real world. Chin. J. Chin. Mater. Med. 40, 2440–2444. 10.4268/cjcmm20151233 26591539

[B112] Yu Y. Cong Y. Quan X. D. Wei J. (2009). Pharmacodynamics studies of XiYanPing for injection. J. Liaoning Univ. Tradit. Chin. Med. 11, 198–200. 10.13194/j.jlunivtcm.2009.07.200.yuy.104

[B113] Yu D. D. Xie Y. M. Liao X. Li H. M. Zhang Y. L. Wang G. Q. (2019). Systematic review and meta-analysis of Huoxiang Zhengqi Pills combined with western medicine for acute gastroenteritis. Chin. J. Chin. Mater. Med. 44, 2914–2925. 10.19540/j.cnki.cjcmm.20190513.502 31602833

[B114] Yu H. X. Xiong L. Hu H. Z. Yang S. C. (2020). The influence of Shufeng Jiedu capsule on the inflammation related factors for the patients with chronic obstructive pulmonary disease in the acute exacerbation. Chin. Med. Mod. Distance Educat. Chin. 18, 61–63. 10.3969/j.issn.1672-2779.2020.01.025

[B115] Yu P. Li Y. Z. Wan S. B. Wang Y. (2020). Observation on the therapeutic effect of Lianhua Qingwen granule combined with abidole in the treatment of mild COVID-19. Chin. Pharma. J. 55, 1–9.

[B116] Zhang H. J. Dong X. L. (2014). The effect of Angong Niuhuang Pill in the treatment of children viral encephalitis. Chin. Pediatr. Integr. Tradit. West. Med. 6, 326–328. 10.3969/j.issn.1674-3865.2014.04.017

[B117] Zhang L. L. Wang G. L. (2015). The study of Xiyanping injection for sever pneumonia in elderly patient. J. Emerg. Tradit. Chin. Med. 24, 2289–2290. 10.3969/j.issn.1004-745X.2015.12.082

[B118] Zhang S. W. Sun C. D. Wen Y. Yin Z. H. (2006). Effect of treatment with Xuebijing injection on serum inflammatory mediators and Th1/2 of spleen in rats with sepsis. Chin. Crit. Care Med. 18, 673–676. 10.1007/s00034-004-1208-7 17092419

[B119] Zhang D. Huang P. Li J. Hu Z. Y. Wang Y. L. (2009). Effects of Angong Niuhuang Boluson vital organ injury and mortality in rats with sepsis. J. Guangzhou Univ. Tradit. Chin. Med. 26, 543–545. 10.3969/j.issn.1007-3213.2009.06.009

[B120] Zhang F. Lu Y. F. Liu J. Shi J. S. (2010). Realgar is active ingredient of Angong Niuhuang Pill in protection against LPS-induced neuro inflammation. Chin. J. Chin. Mater. Med. 35, 3333–3337. 10.4268/cjcmm20102423 21438402

[B121] Zhang Y. Z. Wu H. Y. Ren L. W. Zhang H. S. Jia X. Zhang Y. Z. (2010). Study on modified Shengmai Yin Injection for prevention and treatment of brain impairment in endotoxin shock rats. J. Tradit. Chin. Med. 30, 272–277. 10.1016/S0254-6272(10)60055-6 21287784

[B122] Zhang Z. L. Tong X.,. W. Xu B. Zhu J. (2014). The protective effect of Xuebijing on lipopolysaccharide-induced acute lung injury in rats. Chin. J. Clin. Healthc. 17, 373–375. 10.3969/J.issn.1672-6790.2014.04.014

[B123] Zhang N. Liu J. H. Qiu Z. L. Ye Y. P. Zhang J. Luo T. Z. (2017). Shenfu injection for improving cellular immunity and clinical outcome in patients with sepsis or septic shock. Am. J. Emerg. Med. 35, 1–6. 10.1016/j.ajem.2016.09.008 28029485

[B124] Zhang N. Wang L. L. Deng X. Q. Liang R. Y. Su M. He C. (2020). Recent advances in the detection of respiratory virus infection in humans. J. Med. Virol. 92, 408–417. 10.1002/jmv.25674 31944312 PMC7166954

[B125] Zhang H. K. (2013). Studies on antibacterial material basis and quality control of Huoxiang Zhengqi Tincture (Guangzhou: Guangdong Pharmaceutical College).

[B126] Zhao P. Yang H. Z. Lv H. Y. Wei Z. M. (2014). Efficacy of Lianhuaqingwen Capsule compared with oseltamivir for influenza a virus infection: a meta-analysis of randomized, controlled trials. Altern. Ther. Health Med. 20, 25–30. 24657957

[B127] Zhao W. Li Q. Zhang S. S. Fu Y. J. Shi X. Q. (2014). The effect of Xuebijing injection on immune regulation in SIRS patients. J. Sichuan Univ. (Med. Sci. Edi.) 45, 863–865. 10.13464/j.scuxbyxb.2014.05.032 25341356

[B128] Zhao H. J. Guo L. P. Yang F. W. Zhang M. Y. Zhang L. S. Liu Z. (2017). Huoxiang Zhengqi Formulas for treatment of gastrointestinal type cold:a systematic review and meta-analysis. Chin. J. Chin. Mater. Med. 42, 1495–1499. 10.19540/j.cnki.cjcmm.2017.0047 29071852

[B129] Zheng J. S. Gu L. G. (2009). Study on anti-virus effect of Tan Re Qing injection on the mice with influenza virus FM1 infected. Chin. J. Tradit. Chin. Med. Pharm. 24, 851–854.

[B130] Zhong Y. Q. Mao B. Wang G. Fan T. Liu X. M. Diao X. (2010). Tanreqing injection combined with conventional western medicine for acute exacerbations of chronic obstructive pulmonary disease: a systematic review. J. Altern. Complement. Med. 16, 1309–1319. 10.1089/acm.2009.0686 21091297

[B131] Zhu K. J. Sun J. N. (2014). Study on effect and mechanism of cinnabaris and realgar in promoting awake of endotoxin-induced brain injury rat applied with Angong Niuhuang Wan. Chin. J. Chin. Mater. Med. 39, 4007–4012. 10.4268/cjcmm20142021 25751954

[B132] Zhu M. J. Zhang G. Hu M. H. Chen Y. B. Ji C. L. (2014). Stasis-resolving and detoxifying effect of Xuebijing Injection on severe pneumonia: a systematic review. Chin. J. Evid. Based. Med. 14, 462–468. 10.7507/1672-2531.20140080

[B133] Zhu X. Y. Xie H. F. Han H. Gao F. (2019). Network Meta-analysis of 3 kinds of Chinese medicine injections in treatment of acute exacerbations of chronic obstructive pulmonary disease. Chin. J. Chin. Mater. Med. 44, 2179–2184. 10.19540/j.cnki.cjcmm.20181217.003 31355578

[B134] Zhuo Z. L. Wen Z. F. (2017). Study on effect of Angong Niuhuang pill combined with ribavirin in treatment of acute severe viral pneumonia in children in serum calcitonin and immune function. Liaoning J. Tradit. Chin. Med. 44, 2314–2317. 10.13192/j.issn.1000-1719.2017.11.024

[B135] Zong S. B. Lv Y. Z. Sun L. Li Z. Q. Zhou J. Bi Y. A. (2015). Effect of Jiawei Huoxiang Zhengqi soft capsule on chemotactic cytokine of colitis in rats. *Pharm* . Clin. Res. 23, 23–25. 10.13664/j.cnki.pcr.2015.03.005

