# Supplementary material for: Chinese Patent Medicines in the Treatment of Coronavirus Disease 2019 (COVID-19) in China
Source: Front Pharmacol. 2020 Jul 17;11:1066. doi: 10.3389/fphar.2020.01066 (PMC7396557; doi:10.3389/fphar.2020.01066)
Supplement: Supplementary file 1 [file Table_1.docx]

**Supplementary Table - Basic information on the top 10 CPMs for the treatment of COVID-19**

| **Drug name** | **Ingredients** | **Effects and Indications** | **Usage and Dosage** | **Adverse reactions** | **Cautions** |
| --- | --- | --- | --- | --- | --- |
| HXZQC | *Pogostemon cablin* (Blanco) Benth., *Atractylodes macrocephala* Koidz., *Magnolia officinalis* Rehder & E.H.Wilson, *Pinellia ternata* (Thunb.) Makino, *Perilla frutescens* (L.) Britton, *Angelica dahurica* (Hoffm.) Benth. & Hook.f. ex Franch. & Sav., *Citrus × aurantium* L., *Poria cocos* (Schw.) Wolf, *Platycodon grandiflorus* (Jacq.) A.DC. (Jiegeng), *Glycyrrhiza uralensis* Fisch. ex DC., *Ziziphus jujuba* Mill., *Areca catechu* L. and [*Zingiber officinale* Roscoe](https://mpns.science.kew.org/mpns-portal/plantDetail?plantId=273361&query=Shengjiang&filter=&fuzzy=false&nameType=all&dbs=wcs)*.* | Release the exterior and remove dampness, rectify qi and harmonize the center. In clinical observation period of COVID-19 and early stage of the disease (mild case), and the symptoms are weakness, headache and dizziness, abdominal fullness and distention, vomiting and diarrhea, etc. | Oral administration, four capsules once, two times a day. | Drug eruption, purpura, shock, asthma, intestinal obstruction, upper gastrointestinal hemorrhage, hypoglycemia of childhood, infantile convulsions, etc. | (1) It’s not advisable to take nourishing traditional Chinese medicines during the period of medication. (2) It is advisable to be on light diet during the period of medication. |
| LHQWC | *Forsythia suspensa* (Thunb.) Vahl, *Lonicera japonica* Thunb., *Ephedra equisetina* Bunge, *Prunus armeniaca* L., Gypsum fibrosum, *Isatis tinctoria* L., *Dryopteris crassirhizoma* Nakai, *Houttuynia cordata* Thunb., *Pogostemon cablin* (Blanco) Benth., *Rheum palmatum* L., *Rhodiola rosea* L., Mentholum and *Glycyrrhiza uralensis* Fisch. ex DC. | Clear epidemic and resolve toxins, diffuse the lung and discharge heat. In the clinical observation period of COVID-19 and early stage of the disease (mild case), the symptoms are fever, mild aversion to cold, cough, weakness, headache and body pain, sore throat and constipation. | Oral administration, four capsules once, three times a day. | Nausea, vomiting, diarrhea, stomach discomfort, heat-burn, poor appetite and other gastrointestinal adverse reactions; there might be abnormal liver function, palpitation or rash and other side effects occasionally. | (1) Pregnant and lactating women should use with caution. (2) It contains ephedra, therefore, athletes and patients with high blood pressure and heart disease should use with caution. (3) Those with previous history of liver disease or with abnormal liver function before administration should use with caution. (4) It contains rheum, therefore, those having increased stool frequency and shapeless stool after administration should reduce the dose appropriately. (5) Nourishing traditional Chinese medicine should not be taken at the same time. |
| JHQGG | *Forsythia suspensa* (Thunb.) Vahl, *Lonicera japonica* Thunb., *Ephedra equisetina* Bunge, *Prunus armeniaca* L., Gypsum Fibrosum, [*Scutellaria baicalensis* Georgi](https://mpns.science.kew.org/mpns-portal/plantDetail?plantId=188938&query=Huangqin&filter=&fuzzy=false&nameType=all&dbs=wcs) , *Fritillaria thunbergii* Miq., *Anemarrhena asphodeloides* Bunge, *Arctium lappa* L., *Artemisia annua* L., *Mentha canadensis* L. and *Glycyrrhiza uralensis* Fisch. ex DC. | Scatter wind and diffuse the lung, clear heat and resolve toxins. In the clinical observation period of COVID-19 and early stage of the disease (mild case), and the symptoms are fever, mild aversion to cold, weakness, cough, headache and body pain, and sore throat. | Take after dissolving in boiled water, one bag once, three times a day. | Nausea, vomiting, diarrhea, stomach discomfort, heartburn, poor appetite and other gastrointestinal adverse reactions; there might be abnormal liver function, palpitation or rash occasionally. | (1) Those with deficiency-cold in the spleen and stomach should use with caution. (2) It contains ephedra, therefore, athletes and patients with high blood pressure and heart disease should use with caution. (3) Those with previous history of liver disease or with abnormal liver function before administration should use with caution. (4) Nourishing traditional Chinese medicine should not be taken at the same time. |
| SFJDC | *Reynoutria japonica* Houtt., *Forsythia suspensa* (Thunb.) Vahl, *Isatis tinctoria* L., *Bupleurum chinense* DC., *Patrinia scabiosifolia* Link, *Verbena officinalis* L., *Phragmites australis* (Cav.) Trin. ex Steud. and *Glycyrrhiza uralensis* Fisch. ex DC. | Scatter wind and clear heat, resolve toxins and relieve sore throat. In the clinical observation periods of COVID-19 early stages of the disease (mild case), and the symptoms are fever, aversion to cold, cough with yellow phlegm, weakness and sore throat. | Oral administration, four capsules once, three times a day. | Nausea. | (1) Those with allergic constitution or allergic to the drug are forbidden to use. (2) Those with deficiency-cold in the spleen and stomach are forbidden to use. |
| TRQI | [*Scutellaria baicalensis* Georgi](https://mpns.science.kew.org/mpns-portal/plantDetail?plantId=188938&query=Huangqin&filter=&fuzzy=false&nameType=all&dbs=wcs), Ursi fellis pulvis, *Forsythia suspensa* (Thunb.) Vahl and *Lonicera japonica* Thunb. | Clear heat, dissolve phlegm and resolve toxins. In the progressive stage of COVID-19 (critical case), the symptoms are fever, cough, cough with difficulty in expectoration, chest distress and shortness of breath. | Twenty mL once for adults, 40 mL once for severe patients, with addition of 5% glucose injection or 0.9% sodium chloride injection of 250-500 mL, intravenous drip, and the drop number should be controlled less than 60 drops per min, once a day. | Some patients may have dizziness, chest distress, nausea, vomiting and diarrhea. There are flushing, rash or itching and other allergic reactions occasionally. There is rare palpitation, chill and difficulty in breath. There is extremely rare allergic shock. Other adverse reactions: dry mouth, fever, periorbital facial edema, discomfort in the infusion site, etc. | (1) Those with liver and renal failure are forbidden from use. (2) Those with severe lung and heart disease accompanied by heart failure are forbidden from use. (3) Pregnant women and infants less than 24 months are forbidden from use. (4) It should be used alone, and is forbidden from being mixed with other drugs. (5) Dilution ratio of the liquid should be no lower than 1:10 (liquid: solvent), and the diluted liquid must be used within 4 h. |
| XYPI | Andrographolide total sulfonate | Clear heat and resolve toxins, relieve cough and dysentery. In the progressive stage of COVID-19 (critical case), and the symptoms are fever, sore throat, cough with yellow phlegm and chest distress. It could also be used to treat virus infection combined with mild bacterial infection. | Intramuscular injection. Adults: 50–100 mg once, two or three times a day. Intravenous drip. Adults: 250-500 mg a day, diluted with 0.9% sodium chloride injection or 5% glucose injection. | Rash, itching, shivering, facial blushing, fever, cyanosis, difficulty in breathing, nausea, vomiting, palpitation, chest distress, allergic shock, etc. | (1) Pregnant women and children under 1 year of age are forbidden from use, elderly above 75 years of age should use with caution. (2) Those with a history of allergic or severe adverse reactions to this drug or preparations containing Andrographolide total sulfonate are forbidden from use. (3) It is recommended to enhance monitoring of the patients using XYPI for the first time; pay close attention to the reactions during the administration process, especially if discovering abnormalities within 30 min from administration, stop the administration immediately and take active rescue measures. (4) When in combined use with other injections, XYPI should be dripped first, and then other injections could be infused after flushing the infusion tube or replacing the infusion tube. |
| XBJI | *Carthamus tinctorius* L., *Paeonia lactiflora* Pall., *Conioselinum anthriscoides* 'Chuanxiong', *Salvia miltiorrhiza* Bunge and *Angelica sinensis* (Oliv.) Diels. | Dissolve stasis and resolve toxins. In the progressive stage of COVID-19 (critical case), and the symptoms are fever, dyspnea and tachypnea, palpitation, dysphoria, etc. It could also be used for treatment of infection-induced systemic inflammatory response syndrome and multiple-organ dysfunction syndrome during the stage of impaired organ functions. | Intravenous injection. (1) Systemic inflammatory response syndrome: 50 mL plus 0.9% sodium chloride injection of 100 mL for intravenous drip, which should be finished in 30-40 min, twice a day. Three times a day for severe patients. (2) multiple-organ dysfunction syndrome: 100 mL plus 0.9% sodium chloride injection of 100 mL for intravenous drip, which should be finished in 30-40 min, twice a day. Three or four times a day for severe patients. | Allergic reactions: Skin flush, rash, itching, palpitation, cyanosis, laryngeal edema, allergic shock, etc. Cardiovascular system: palpitation, cyanosis, increase or decrease of blood pressure, arrhythmia. Nervous system: Dizziness, headache. Respiratory system: difficulty in breathing, chest distress, labored breathing, short breath, cough. Digestive system: nausea, vomiting, stomach pain, diarrhea, abnormal liver function. Others: facial edema, conjunctival congestion, abnormal tears, phlebitis, lumbago, backache, local numbness. | (1) Pregnant women and children under 14 (included) years of age are forbidden from use. (2) The product is forbidden from being mixed with others, and should be used in combination with other drugs with caution, when in combined use with other drugs, 50 mL 0.9% sodium chloride injection shall be used for the intervals. (3) Allergic history, family allergic history and history of medications of the patients should be inquired before administration. (4) During administration, pay special attention to the initial 30 min of intravenous drip, and in case of an abnormality, discontinue the drug immediately and conduct symptomatic treatments. (5) Monitoring of the administration shall be enhanced toward elder patients and the patients using TCM injection for the first time. |
| SFI | *Panax ginseng* C.A.Mey. and *Aconitum carmichaeli* Debeaux. | Restore yang to rescue from counterflow [desertion], boost qi to rescue from desertion. In the progressive stage of COVID-19 (critical case), and the symptoms are dyspnea, pale complexion, and the severe symptoms are unconsciousness, drip sweat, cold limbs. | Intravenous drip: 20-100 mL once, diluted in 5%-10% glucose. Injection of 250-500 mL. Intravenous injection:  5-20 mL once, diluted in 5%-10% glucose Injection of 20 mL. | Dizziness, headache, shivering, fever, palpitation, chest distress, chest pain, difficulty in breathing, nausea, retching, abdominal pain, rash, itching, rash or swelling, pain and other discomfort in local infusion sites. | (1) Pregnant women should use with caution. (2) Avoid being directly mixed with coenzyme A, VitK3 and aminophylline. (3) Prepared drug should be used within 4 h. |
| SMI | *Panax ginseng* C.A.Mey. and *Ophiopogon japonicus* (Thunb.) Ker Gawl. | Boost qi to rescue from desertion, nourish yin and promote fluid production, and engender pulse. In the progressive stage of COVID-19(critical case), and the symptoms are weakness and shortness of breath, tachypnea, palpitation, dry mouth, sweating, and even dysphoria and cold limbs. | Intravenous drip, 20-60 mL each time, diluted in 5% glucose. Injection of 250-500 mL. | Itching, rash and systematic urticarial, and then allergic shock; abdominal distension, corneal edema, abnormal vision, hypotension, ascending vascular pain, acute hepatic damage, sinus arrest and drug fever. | (1) Newborns, infants, pregnant women and those with allergic constitution are forbidden from use. (2) Administrative method of intravenous injection should not be used, the administration speed cannot be too quick, and for those using the drug for the first time, observe 10 min at the rate of 15 drips/min initially, and in case of no abnormalities, then drip at normal speed, which is generally controlled at 40-50 drips/min. (3) This drug has a suppressor response, and the hypertension patients should pay attention to changes in blood pressure during use. |
| AGNHP | *Curcuma kwangsiensis* S.G.Lee & C.F.Liang, Calculus Bovis, Cornu Bubali, *Coptis chinensis* Franch., Cinnabaris, Moschus, Margarita, Realgar, [*Scutellaria baicalensis* Georgi](https://mpns.science.kew.org/mpns-portal/plantDetail?plantId=188938&query=Huangqin&filter=&fuzzy=false&nameType=all&dbs=wcs), *Gardenia jasminoides* J.Ellis and *Cinnamomum camphora* (L.) J.Presl. | Clear heat and resolve toxins, suppress fright and open the orifices. In the progressive stage of COVID-19 (critical case), and the symptoms are hyper-pyretic convulsion, coma and delirium, difficulty in breathing, dysphoria, etc. | Oral administration, 3 g once, once a day. | Overdose administration might cause mercurial nephrosis or allergic reaction and other adverse reactions, and improper use of this product might cause hypothermia. | (1) Those who cannot take it orally due to high fever and coma, could be made nasogastric administration after dissolving in warm water(40-60℃), and do not dissolve the pills with hot water. (2) It is forbidden from taking together with nitrate, nitrite, ferrite and sulfate drugs. (3) Pregnant women are forbidden from use. (4) It contains cinnabar and realgar, and cannot be taken as an overdose for a long time, and those with hepatic and renal dysfunction should use it with caution. (5) It contains musk, and athletes should use it with caution. |
